# RNAi-mediated knockdown of the poultry red mite cathepsin D-1 impacts haemoglobin digestion

**DOI:** 10.1186/s13071-026-07254-y

**Published:** 2026-02-06

**Authors:** Wan Chen, Naomi R. Defty, Kathryn Bartley, Francesca Nunn, Antonella Schiavone, Alan S. Bowman, Adam D. Hayward, Stewart T. G. Burgess, Alasdair J. Nisbet, Daniel R. G. Price

**Affiliations:** 1 Moredun Research Institute, Pentlands Science Park, Bush Loan, Edinburgh, EH26 0PZ UK; 2 Present Address: Institute of Biological and Environmental Sciences, School of Biological Sciences, University of Aberdeen, Aberdeen, AB24 3FX UK; 3 Present Address: School of Life Sciences, Key Laboratory of Jiangxi Province for Biological Invasion and Biosecurity, Jinggangshan University, Ji’an, Jiangxi China

**Keywords:** Haematophagous, Ectoparasite, Lysosomal proteinase, Aspartyl proteinase, Cathepsin D

## Abstract

**Background:**

The poultry red mite, *Dermanyssus gallinae*, is a haematophagous ectoparasite causing significant economic losses in the commercial egg-laying sector. Blood meal digestion by *D. gallinae* is required for nutrient acquisition, with acidic lysosomal proteinases such as cathepsin L and cathepsin D playing a critical role in haemoglobin digestion. This study investigated the role of a cathepsin D-like aspartyl proteinase, Dg-CatD-1, in the haemoglobin digestion cascade.

**Methods:**

Haemoglobin processing was investigated by RNA interference (RNAi)-mediated silencing of *Dg-CatD-1* and assessing the impact on haemoglobin digestion. RNAi-mediated knockdown of *Dg-CatD-1* was achieved by feeding a target-specific double-stranded RNA (dsRNA) to *D. gallinae* in a blood meal. The minimum length and concentration of *Dg-CatD-1* dsRNA for effective knockdown was determined. In addition, the effect of *Dg-CatD-1* knockdown on mite digestive physiology, haemoglobin digestion, and egg-laying by adult female mites was assessed.

**Results:**

Feeding *Dg-CatD-1* dsRNAs via a blood meal to adult female *D. gallinae* mites resulted in a substantial knockdown of target gene expression. The minimum length and concentration of dsRNA required for effective *Dg-CatD-1* knockdown were 25 base pairs (bp, at 200 ng/μl) (61% knockdown) and 25 ng/μl (at 485  bp) (42% knockdown), respectively. When *Dg-CatD-1* dsRNA was delivered as a single feed it resulted in up to 91% reduction in *Dg-CatD-1* expression, although no observable effect on blood digestion was observed. The phenotypic impact of *Dg-CatD-1* knockdown was demonstrated following two consecutive rounds of *Dg-CatD-1* dsRNA feeding where knockdown reduced the ability of mites to process and clear their blood meal relative to control non-specific dsRNA-fed mites.

**Conclusions:**

This work highlights the importance of Dg-CatD-1 as an essential enzyme in the haemoglobin digestion pathway of *D. gallinae*. These findings open avenues for the development of targeted control strategies aimed at disrupting the digestive processes of *D. gallinae*. Furthermore, this research suggests that reductions in gene expression via RNAi do not always lead to corresponding decreases in protein levels or observable phenotypes. Repeated exposure to dsRNA may be necessary to reveal phenotypic effects of gene knockdown.

**Graphical Abstract:**

**Figure:**
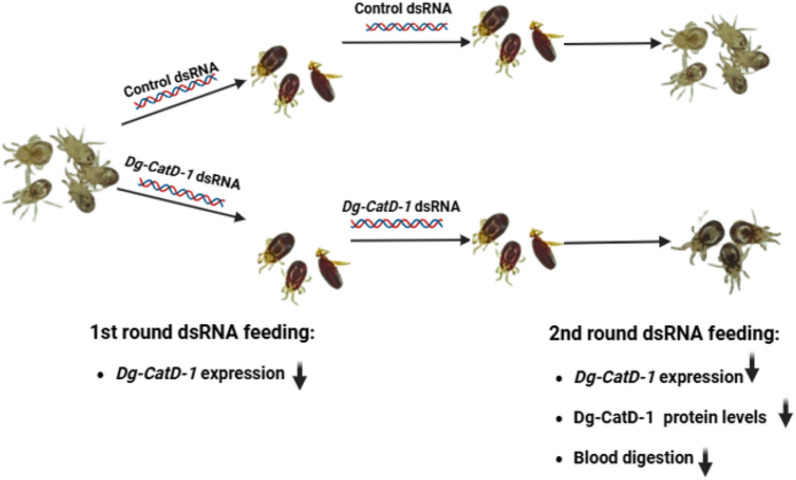

**Supplementary Information:**

The online version contains supplementary material available at 10.1186/s13071-026-07254-y.

## Background

Haemoglobin is a key nutrient, playing a crucial role in providing the resources required for embryogenesis and oviposition in haematophagous arthropods [1]. The haematophagous poultry red mite, *Dermanyssus gallinae*, is an ectoparasite which feeds on avian blood and causes major welfare concerns and economic losses in the poultry sector [2, 3]. Three of the five *D. gallinae* life stages (protonymphs, deutonymphs, and adults) are blood-feeding, with mites able to rapidly process their blood meal [4]. Digestion of haemoglobin plays a key role in the nutritional physiology of *D. gallinae*, and previous studies have demonstrated that haemoglobin digestion by soluble *D. gallinae* extracts is most efficient at acidic pH [5, 6], with digestion being inhibited by E-64 and pepstatin A, indicating roles for cysteine and aspartyl proteinases, respectively [5]. Consistent with these observations, transcriptomic analysis of blood-feeding stages of *D. gallinae* has identified upregulation of gut-specific cysteine and aspartyl proteinases (cathepsin D-like and cathepsin L-like) in response to blood-feeding [7, 8].

In ticks, which are the most extensively studied among blood-feeding Acari, blood digestion occurs in enclosed compartments within specialised digestive cells. Within these cells, blood protein digestion takes place within acidic digestive vesicles (secondary lysosomes). In the tick haemoglobin digestion cascade, haemoglobin is initially cleaved via lysosomal endopeptidases: aspartyl proteinase cathepsin D (CatD), cysteine proteinase cathepsin L (CatL) [9, 10], and the asparaginyl endopeptidase legumain (AE) [11]. The biological and phylogenetic similarities between *D. gallinae* and soft ticks (Argasidae) have led to the assumption that, in *D. gallinae*, after erythrocyte lysis, blood meal digestion takes place intracellularly and occurs in acidic secondary lysosomes [5, 12, 13]. Thus, like ticks, *D. gallinae* may use an intracellular digestion strategy to process host haemoglobin, where degradation starts with haemoglobin uptake into endosomes in gut digestive cells, followed by processing via a multi-proteinase cascade in secondary lysosomes formed by their fusion with membrane-bound intracellular material after phagocytosis [9, 14, 15].

In blood-feeding parasites, aspartyl proteinases have a key role in this multi-proteinase cascade of digestion, either in initiating haemoglobin hydrolysis or in activating other proteinases, and have consequently attracted attention as potential targets for vaccination as well as other pharmaceutical interventions [9, 16]. In *D. gallinae*, expression of a gene encoding a cathepsin D-like aspartyl proteinase, *Dg-CatD-1*, was increased in blood-fed adult female mites [7]. Consistent with its essential role, mites that ingested blood containing antibodies against recombinant Dg-CatD-1 exhibited over four times greater mortality than controls [7]. More recently, in 2019, Price et al. demonstrated that *D. gallinae* adult female mites feeding on hens immunised with recombinant Dg-CatD-1 laid significantly fewer eggs than mites feeding on unimmunised hens [6]. Both vaccine effects, increased mortality and reduced egg-laying, associated with immunisation with Dg-CatD-1 have been independently confirmed [17].

Given the growing evidence for the role of Dg-CatD-1 in blood meal digestion and its potential as a vaccine candidate, we developed a gene-silencing strategy to study the effects of its transcriptional suppression. Due to the likely importance of Dg-CatD-1 in blood meal digestion, we hypothesised that the protein may be so abundantly expressed prior to gene silencing that, combined with a low turnover rate, a masked phenotype could result. In this scenario, relatively high levels of residual protein would persist even after messenger RNA (mRNA) expression has been effectively suppressed. This led us to design a “double-silencing” experiment, where mites are exposed to two consecutive RNA interference (RNAi) treatments prior to phenotype assessment. This has allowed us to examine the phenotypes associated with *Dg-CatD-1* knockdown to better understand its biological function and importance.

## Methods

### Bioinformatics analysis of *Dg-CatD-1*

To identify the *D. gallinae* genes encoding cathepsin D-like aspartyl proteinases, we used the Basic Local Alignment Search Tool (BLAST), employing the previously described *Dg-CatD-1* nucleotide sequence (accession no. HE565350) as a query in blastn and blastx searches (using a BLOSUM62 matrix) against coding sequences and proteins, respectively, from the *D. gallinae* genome. Searches were conducted at OrCAE [18] which hosts the *D. gallinae* genome [19]. Functional domains were identified in all retrieved sequences using InterProScan [20]. The *D. gallinae* aspartyl protease active site motifs (DTG/DSG) were identified using InterPro domain predictions (e.g., InterPro aspartic peptidase, active site, IPR001969) and confirmed by sequence alignment with the well-annotated Dg-CatD-1 reference sequence (HE565350) as described by Bartley et al., 2012 [7].

### *Dermanyssus gallinae* collection and conditioning

Mixed-life-stage *D. gallinae* were collected from a commercial organic egg-laying poultry farm in the Scottish Borders, UK. Collected mites were kept for 7 days at 4 °C in Corning^®^ 75-cm^2^ cell culture canted neck flasks, then transferred to an MLR-351H incubator (Sanyo, Osaka, Japan) at 20–22 °C with 75% relative humidity (RH) and maintained in darkness for an additional 7 days before RNAi gene-silencing studies.

### dsRNA synthesis

Double-stranded RNA (dsRNA) representing two separate regions of the *Dg-CatD-1* gene were synthesised. Region 1 (R1: 357 base pairs [bp], corresponding to exon 5) and region 2 (R2: 485 bp, corresponding to exons 6–8) of the *Dg-CatD-1* gene (accession no. HE565350) were amplified from complementary DNA (cDNA) generated from adult female *D. gallinae* using Phusion proofreading polymerase (Thermo Fisher Scientific, Waltham, MA, USA). Each forward and reverse primer contained an NcoI and NheI restriction enzyme site, respectively, to allow directional cloning into the RNAi vector pL4440 (pL4440 was a gift from Andrew Fire [Addgene plasmid # 1654; http://n2t.net/addgene:1654]). Primer sequences are shown in Additional file 1: Table S1. Amplification products for *Dg-CatD-1* R1 (357 bp) and R2 (485 bp) were digested with NcoI and NheI and cloned into the corresponding restriction enzyme sites of pL4440. Plasmids were used to transform chemically competent *Escherichia coli* JM109 cells (Promega, Madison, WI, USA), and plasmid was isolated from *E. coli* transformants using a Wizard^®^ Plus SV Minipreps DNA Purification System (Promega, Madison, WI, USA). RNAi constructs containing *Dg-CatD-1* R1 and R2 were verified by Sanger sequencing. For control (non-target) dsRNA production, a previously generated construct containing a region of the *E. coli* strain K-12 *lacZ* gene NC_000913 (319 bp; 63–381 bp of the coding sequence [CDS]) cloned into the SacI and SmaI sites of pL4440 was used [21]. The dsRNAs were synthesised using the T7 RiboMAX Express RNAi System (Promega, Madison, WI, USA), according to the manufacturer’s instructions. For dsRNA synthesis, *Dg-CatD-1* pL4440 plasmids were linearised with either NcoI or NheI for sense or antisense transcription, respectively. Control *lacZ* pL4440 plasmid was linearised with SmaI or BglII for sense or antisense transcription, respectively. For dsRNA production, equimolar amounts of complementary RNAs were mixed and incubated at 70 °C, then slowly cooled to room temperature to allow annealing. Annealed dsRNAs were treated with DNAse and RNAase, then purified by sodium acetate/isopropanol precipitation and resuspended in nuclease-free water. Purified dsRNAs were analysed by agarose/TAE (Tris–acetate–EDTA [ethylenediaminetetraacetic acid]) gel electrophoresis to confirm quality and quantified on a NanoDrop One spectrophotometer (Thermo Fisher Scientific, Waltham, MA, USA).

**Fig. 1:**
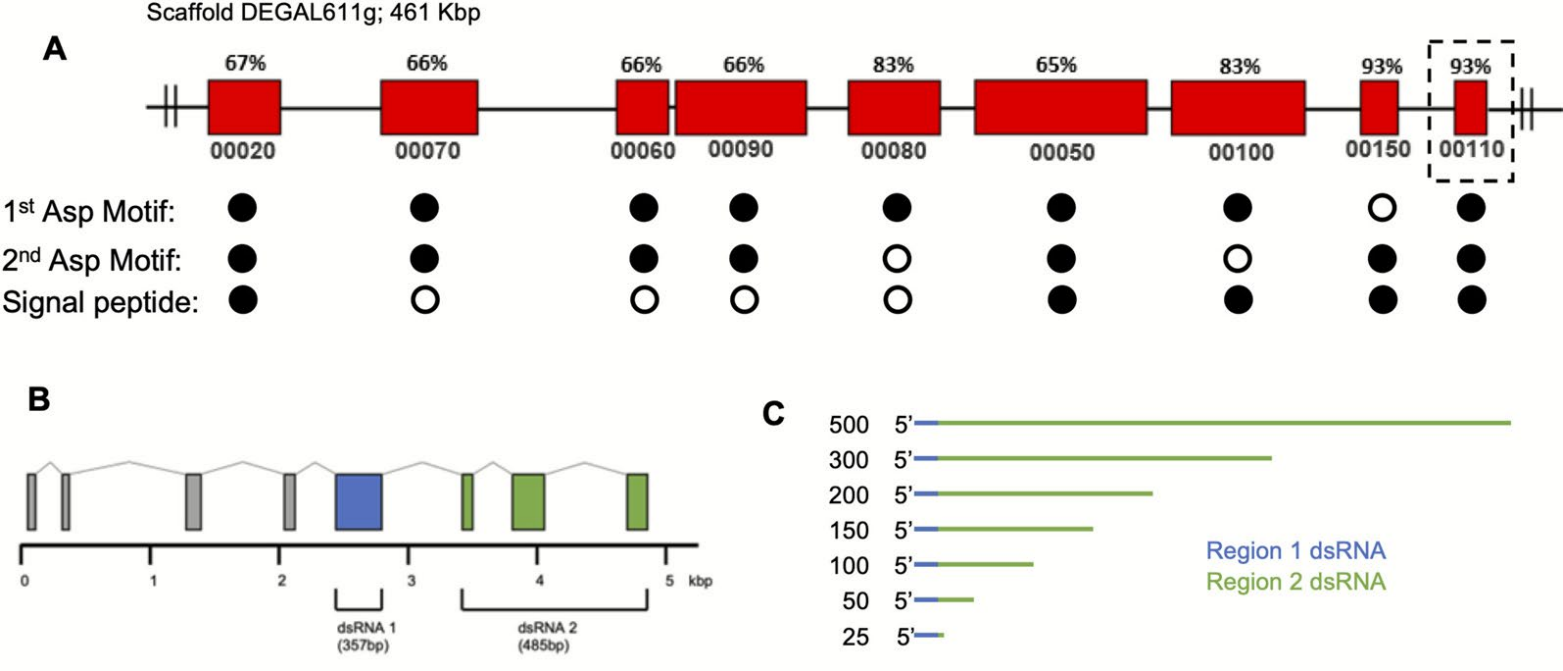
Schematic overview of the genomic region encoding the *D. gallinae* cathepsin D-1 (*Dg-CatD-1*) gene and regions used for dsRNA synthesis. **A** Overview of the 204-kbp region of *D. gallinae* genomic scaffold 611 containing the *Dg-CatD-1* coding region and related paralogs. The sequence with highest nucleotide identity to *Dg-CatD-1* (HE565350) is highlighted, and the percentage nucleotide identity to each of the paralogs is shown. For each gene, the unique part of the sequence identifier is shown; for the full gene name, append DEGAL611g, e.g., DEGAL611g00110. For each encoded cathepsin-D like aspartyl proteinase, conserved catalytic aspartic acid residues and predicted signal peptides are indicated (closed circles indicate presence of sequence feature). **B** Schematic representation of the *Dg-CatD-1* genomic DNA (gDNA) locus for DEGAL611g00110 highlighting the location of two regions that were used for dsRNA design. Exons are shown as boxes and introns as interconnecting lines; dsRNA region 1 (357 bp, exon 5) indicated by the blue box, and dsRNA region 2 (485 bp, exons 6–8) indicated by the green boxes. **C** For RNAi length experiments, a series of overlapping dsRNAs were constructed; the long 500-bp dsRNA included 15 bp from region 1 (highlighted in blue) and 485 bp from region 2 (500 bp in total). The regions used to construct the shorter fragments (ranging from 300 to 25 bp) are shown

For *Dg-CatD-1* dsRNA studies to determine the optimal and minimum lengths of dsRNA for effective gene silencing, template DNA for RNA transcription was generated by polymerase chain reaction (PCR) using primers that incorporated 5′ and 3′ T7 RNA polymerase binding sites. Overlapping dsRNAs were synthesised, ranging from 25 to 500 bp in length, from regions 1 and 2 of the *Dg-CatD-1* gene (Fig. 1C). Primer sequences are shown in Additional file 1: Table S1. Each region was amplified from a plasmid containing the full-length coding sequence for *Dg-CatD-1* using Platinum™ Taq DNA polymerase (Thermo Fisher Scientific, Waltham, MA, USA). Amplicons were analysed by agarose/TAE gel electrophoresis to confirm quality and predicted size. The remaining reaction was purified using the Wizard^®^ SV Gel and PCR Clean-Up System (Promega, Madison, WI, USA) and quantified using a NanoDrop One spectrophotometer (Thermo Fisher Scientific, Waltham, MA, USA). For dsRNA synthesis, purified DNA was used as a template for in vitro transcription reactions, and dsRNA was quantified and validated as described above. For the synthesis of the shortest—25-bp—dsRNA, overlapping DNA oligonucleotides containing T7 RNA polymerase binding sites were annealed to create a double-stranded DNA (dsDNA) template. The sequence of overlapping oligonucleotide sequences (oligos) is shown in Additional file 1: Table S1. In brief, oligos sense 1 and sense 2, and separately, oligos antisense 3 and antisense 4, were annealed to create a template for sense and antisense transcription, respectively. For dsRNA synthesis, annealed oligos were used in transcription reactions according to the manufacturer's protocols supplied with the T7 RiboMAX Express RNAi System (Promega, Madison, WI, USA) and quantified and validated as described above.

### Oral delivery of target gene dsRNA by in vitro feeding

#### In vitro feeding of adult female *D. gallinae*

Blood-feeding of adult female *D. gallinae* was conducted using *in vitro* feeding devices with a baudruche (goldbeaters cloth) membrane, as described by Nunn et al. [22]. In line with the 3R principles (Replacement, Reduction, and Refinement), and as a refinement to the procedure, the feeding devices contained goose blood instead of hen blood, reducing the number of procedures (bleeds) required to obtain sufficient material for feeding assays. Previous work has demonstrated the suitability of goose blood for in vitro feeding, with comparable levels of mite feeding, survival, and egg-laying observed on both hen and goose blood [22]. Following feeding, the level of *D. gallinae* engorgement was assessed by microscopy, and mites that were fully engorged (approximately double in size relative to unfed) and with a bright red blood meal that was clearly visible through the transparent cuticle were selected for monitoring.

#### Single-administration dsRNA feeding experiments

Adult female mites were selected from a mixed population based on their morphology, as female *D. gallinae* are much larger than both males and other life stages and can be further identified by their epigynous shield. These mites were placed into replicate in vitro feeding devices (as described in Nunn et al. [22]), with 70–75 mites per device. A minimum of three replicate devices were used for each experimental treatment or control. These feeding devices containing mites were incubated overnight in darkness in a MLR-351H incubator (Sanyo, Osaka, Japan) at 20–22 °C, 75% RH, before the experiment. For dsRNA feeding, heparinised goose blood (20 units/ml heparin) containing either *Dg-CatD-1* or *lacZ* (control) dsRNA was added to the blood reservoir, with dsRNA at 12.5–400 ng/μl (w/v), depending on the experiment. The concentration of control dsRNA (*lacZ*) was matched to the highest concentration of *Dg-CatD-1* dsRNA delivered in each experiment (400 ng/μl for concentration experiment 1, 200 ng/μl for concentration experiment 2). For optimal dsRNA length experiments, dsRNA (both *Dg-CatD*-1 and *lacZ*) was present in the blood at 200 ng/μl (w/v) (final concentration) and tested at lengths of 500, 300, 200, 150, 100, 50, and 25 bp. Following addition of the blood/dsRNA mixture, all devices were placed in an MLR-351H incubator (Sanyo, Osaka, Japan) for 3 h at 39 °C and 75% RH in darkness for dsRNA oral delivery. The blood-feeding status of the mites was then determined by microscopy (as described above), and all fully engorged mites were recovered into a 1.5-ml Eppendorf tube either covered with AeraSeal™ films (A9224, Sigma-Aldrich, St. Louis, MO, USA) or with the lid pierced using a 26-gauge needle. Approximately 20 mites were placed into each tube and placed back into the incubator at 25 °C and 75% RH for a further 72 h in darkness to allow time for blood meal digestion. All surviving mites were then transferred into new 1.5-ml Eppendorf tubes before snap freezing in liquid nitrogen and storage at −70 °C.

#### Consecutive-administration dsRNA feeding experiments

For consecutive dsRNA feeding experiments, adult female mites were picked and fed the first blood meal containing either *Dg-CatD-1* region 1 dsRNA or *lacZ* dsRNA, each at 200 ng/μl, as described above. At 72 h post-feeding, the mites from each feeding device were split into two groups (group1 and group 2). Group 1 mites were snap frozen in liquid nitrogen and stored for total RNA isolation. Group 2 mites were returned to in vitro feeding devices for a second round of dsRNA feeding, with devices containing either *Dg-CatD-1* region 1 dsRNA or lacZ dsRNA at 200 ng/μl. Mites within the same treatment groups were exposed to blood containing the same dsRNAs (*lacZ* or *Dg-CatD-1*) for both feeding rounds. The blood-feeding status of the mites was then determined by microscopy, and all fed mites were recovered into a 1.5-ml Eppendorf tube either covered by AeraSeal™ films (A9224, Sigma-Aldrich, St. Louis, MO, USA) or with the lid pierced using a 26-gauge needle (approximately 20 mites per tube) and placed back into the incubator at 25 °C and 75% RH for a further 72 h in darkness. All surviving mites were transferred into new 1.5-ml Eppendorf tubes before snap freezing in liquid nitrogen and storage at −70 °C.

### Phenotypic assessment of mites following dsRNA delivery

To assess mite phenotypes following knockdown of *Dg-CatD-1*, blood meal digestion, mite egg production, and mite mortality were measured. Blood meal digestion in *D. gallinae* mites was assessed using a standardised scoring system adapted from that described by Ma et al., 2022 [23]. Blood digestion scores, ranging from 0 (undigested) to 3 (mostly digested) (Fig. 3B), were measured 72 h post-feeding and were based on the intestinal profiles viewed from the dorsal surface, including the shape of the midgut, and caeca I, II, and III. Mite egg production by adult female *D. gallinae* was calculated by monitoring egg numbers 3 days after feeding. Mortality rates of *D. gallinae* were also calculated at 72 h after feeding.

###  Real-time quantitative  PCR 

Real-time quantitative  PCR (RT-qPCR) was used to quantify *Dg-CatD-1* gene expression in adult female mites from the RNAi feeding trials. Total RNA from mites was isolated using the Zymo Research Quick-RNA™ Tissue/Insect RNA Microprep Kit (Zymo Research, Irvine, CA, USA), with the additional DNaseI digestion step, according to the manufacturer’s protocol. When required, protein was recovered from mite lysate (from the same matched RNA extraction) by acetone precipitation, according to the manufacturer’s protocol.

Total RNA was quantified using a NanoDrop One spectrophotometer (Thermo Fisher Scientific, Waltham, MA, USA), and first-strand cDNA was synthesised using the SuperScript IV synthesis kit (Thermo Fisher Scientific, Waltham, MA, USA) with an Oligo(dT)20 primer, according to the manufacturer’s protocol. The final products were diluted 1:5 in nuclease-free water and stored at −20 °C until use.

Primers for RT-qPCR were designed to cover exon–exon junctions were possible to prevent the amplification of genomic DNA, with primers designed outside dsRNA target regions. The qPCR primers were designed for the target gene *Dg-CatD-1* (accession no. HE565350) and three housekeeping reference genes—glyceraldehyde 3-phosphate dehydrogenase (*Dg-GAPDH*; DEGAL4146g00090), apolipophorin-1 (*Dg-apoLp-1*; DEGAL4146g00090), and elongation factor 1 alpha (*Dg-EF-1α-1*; DEGAL4146g00090)—using Primer3Plus [24]. Primer sequences are shown in Additional file 1: Table S1. For construction of standard curves, qPCR primers were used to amplify *Dg-CatD1* (accession no. HE565350), *Dg-GAPDH* (DEGAL4146g00090), *Dg-apoLp-1* (DEGAL4146g00090), and *Dg-EF-1α-1* (DEGAL4146g00090) from adult female *D. gallinae* cDNA. Amplification products were cloned into vector pJET1.2 (Thermo Fisher Scientific, Waltham, MA, USA) and verified by DNA sequencing. Plasmids were used in qPCR experiments to construct standard curves from 10^2^–10^8^ copies of each gene. The qPCR reactions were carried out in a volume of 10 μl comprising 1× PowerUp SYBR Green Master Mix (Thermo Fisher Scientific), 500 nM of forward and reverse primers, and cDNA derived from 1 ng total RNA for each sample. PCR reactions were performed on an Applied Biosystems 7500 Real-Time PCR System (Applied Biosystems, Foster City, CA, USA); thermal cycling conditions were 50 °C for 2 min, then 95 °C for 2 min, followed by 40 cycles of 95 °C for 15 s, 58 °C for 15 s, and 72 °C for 1 min. *Dg-CatD-1* gene expression was normalised to the average of three housekeeping genes (*Dg-GAPDH*, *Dg-apoLp-1*, and *Dg-EF-1α-1*), and expression levels were reported relative to control (*lacZ*) dsRNA-fed mites. The qPCR experiments were performed in triplicate and included “no template” controls and “no reverse transcription” controls with each run.

### Assessment of protein levels of Dg-CatD-1 and haemoglobin post-RNAi-mediated gene knockdown

Proteins precipitated during the RNA purification described above were used for sodium dodecyl sulfate–polyacrylamide gel electrophoresis (SDS-PAGE) and Western blot assay. Briefly, protein samples were prepared in NuPAGE lithium dodecyl sulfate (LDS) sample buffer with reducing agent and heated to 70 °C for 10 min prior to loading on NuPAGE 4–12% Bis–Tris gels. Gels were run in 2-(*N*-morpholino)ethanesulfonic acid (MES) sodium dodecyl sulfate (SDS) running buffer, and proteins were transferred to nitrocellulose membranes by electroblotting in 1× NuPAGE transfer buffer, according to the manufacturer’s instructions (Thermo Fisher Scientific, Waltham, MA, USA). After transfer, to prevent non-specific protein binding, membranes were blocked by incubation in 5% (w/v) milk powder in Tris-buffered saline with Tween 20 [TBST; 20 mM Tris–HCl, pH 7.6, 150 mM NaCl, 0.1% (v/v) Tween^®^ 20] for 2 h at room temperature. The nitrocellulose membranes were cut horizontally at 14 and 17 kDa using a protein molecular weight ladder as a guide. The upper membrane section was incubated with anti-Dg-CatD-1 antibody [6] (used at 1:3000); the middle membrane section was incubated with anti-*D. gallinae* histamine-releasing factor antibody (Dg-HRF-1, accession no. FM179713) [25] (used at 1:100); and the lower membrane section was incubated with anti-chicken haemoglobin beta antibody (LS Bio, USA) used at 1:400. All primary antibodies were diluted in 5% milk powder in TBST and incubated with membrane sections overnight at 4 °C. After washing three times in TBST, membrane sections were incubated with anti-rabbit immunoglobulin G (IgG) [H+L] horseradish peroxidase (HRP) (Thermo Fisher Scientific, MA, USA) at 1:20,000 dilution in 5% milk powder in TBST for 1 h at room temperature. Membranes were washed again, as above, and then immersed in SuperSignal™ West Pico PLUS Chemiluminescent Substrate (Thermo Fisher Scientific), following the manufacturer’s guidelines. Detected bands were imaged using an ImageQuant™ LAS 4000 Biomolecular Imager (GE Healthcare, Sweden), and bands were quantified in ImageJ (version 1.52, National Institutes of Health, USA). For each probed protein, the same-sized rectangular frame was used to detect the density of all individual bands, which were normalised against the background density. Bands from the same loading lane were then normalised to the loading control Dg-HRF-1 from the same lane.

### Statistical analysis

Data from *Dg-CatD-1* transcriptional responses in RNAi oral delivery experiments were plotted in GraphPad Prism (version 9.0, GraphPad Software, USA). For RT-qPCR data, all data were first tested for normality using a Shapiro–Wilk test, which found that the datasets were all normally distributed. Then an unpaired Student’s *t*-test was used for the data with only two variables, where the effect of feeding *Dg-CatD-1* dsRNA from both regions 1 and 2 combined was compared to the *lacZ* control group. A one-way analysis of variance (ANOVA) was used to analyse data with three or more variables, when the effects of feeding either region 1 or 2 Dg-CatD-1 dsRNA, dsRNA length, and dsRNA concentration were compared to a *lacZ* control group. Differences between individual treatments were then analysed using Tukey’s post hoc multiple comparison test for post hoc analysis of the trial using either region 1 or 2 Dg-CatD-1 dsRNA, and Dunnett’s multiple comparison test was used for post hoc analysis of all dsRNA length and concentration experiments. Fecundity data were analysed using two-way ANOVA to check for an effect of both dsRNA type and whether it was the first or second feed on the total number of eggs laid per mite.

Variation in blood meal digestion scoring between the two groups was analysed using ordinal models with the R package “ordinal” [26]. The digestion score was the response variable, with group fitted as a fixed effect and replicate as a random effect. The fixed effect of group was tested using a likelihood ratio test, comparing the model with group and a model without.

## Results

### Cathepsin D is duplicated in the *D. gallinae* genome

The gene encoding *Dg-CatD-1* was previously described (accession no. HE565350) and has a coding sequence of 1152 bp, which translates into a 41.3-kDa polypeptide [7]. Since this initial report, additional *D. gallinae* cathepsin D-like sequences have been identified, with expression of each enriched in the *D. gallinae* adult midgut [8]. Here, by interrogating the *D. gallinae* genome [19], we show that there are nine cathepsin D-like paralogs, which are arrayed on genomic scaffold DEGAL611g (461 kbp). Among these, *Dg-CatD-1* (accession no. HE565350) is most similar to DEGAL611g00150 and DEGAL611g00110 (each with 93% nucleotide identity to HE565350), with nucleotide identity between *Dg-CatD-1* and the remaining paralogs ranging from 65% to 83% (Fig. 1A). Each of these *D. gallinae* cathepsin D-like sequences are members of the aspartic peptidase A1 family (InterPro, IPR001461), and the majority, with the exception of DEGAL611g00080, DEGAL611g00100, and DEGAL611g00150, contain conserved-active-site aspartic acid residues (Fig. 1A and Additional file 2: Figure S1). In addition, five of the encoded proteins contain signal peptides, indicating that these are likely secreted proteinases (Fig. 1A).

Both the amino acid and the nucleotide identity of HE565350 were investigated across all Dg-CatD-1 paralogs, and all sequences were aligned using Clustal Omega (Additional file 2: Figure S1). Gene model DEGAL611g00110 was used to design two regions for dsRNA synthesis; region 1 corresponds to exon 5 (375 bp) and region 2 corresponds to exon 6–8 (485 bp) (Fig. 1B). Across all Dg-CatD paralogs, the nucleotide identity of regions 1 and 2 ranges from 61% to 100%, indicating that the synthesised Dg-CatD-1 dsRNAs may differ in their specificity toward individual paralogs (Additional file 3: Table S2).

### Optimisation of *Dg-CatD-1* silencing

#### Silencing *Dg-CatD-1* using long, gene-specific dsRNAs

**Fig. 2:**
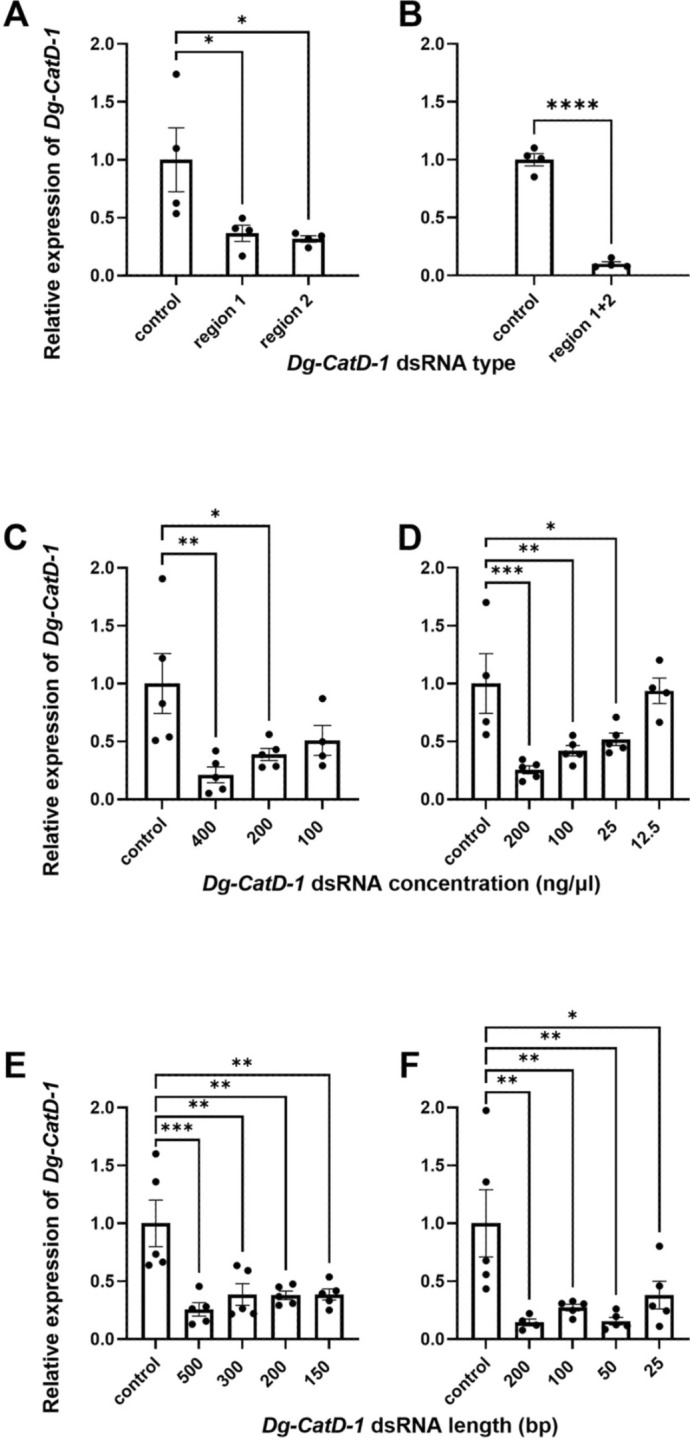
RNAi gene knockdown of *Dg-CatD-1* following oral delivery of target-specific dsRNA. Real-time RT-qPCR analysis of *Dg-CatD-1* expression in adult female *D. gallinae* following oral delivery of either *Dg-CatD-1* or control (*lacZ*) dsRNA in goose blood. **A**, **B** Gene knockdown following oral delivery of dsRNA from regions 1 and 2, with dsRNA fed separately (**A**) or combined (**B**) at a final concentration of 200 ng/μl. **C**, **D** Effect of dsRNA concentration on knockdown of *Dg-CatD-1*. Two separate experiments were conducted (**C**, **D**) using dsRNA from region 2 (485 bp) delivered at final concentrations ranging from 400 to 12.5 ng/μl. **E**, **F** F Effect of dsRNA length on knockdown of *Dg-CatD-1*. Two separate experiments were conducted (**E**, **F**) using *Dg-CatD-1* dsRNA ranging from 500 to 25 bp. Each RNA was delivered at a final concentration of 200 ng/μl. For all experiments, *Dg-CatD-1* expression was monitored at 72 h post-dsRNA delivery and was normalised to the average of either three *D. gallinae* housekeeping genes (*Dg-GAPDH*, *Dg-apoLp-1*, and *Dg-EF-1α-1*) (**A**, **B**) or two housekeeping genes (*apoLp* and *EF-1α*) (**C**, **D**, **E**, **F**). Expression of *Dg-CatD-1* is shown relative to control (*lacZ*-fed) mites. Individual data points for biological replicates are shown with mean ± standard error of the mean (SEM) (*n* = 3–4). Asterisks represent significant differences at *P* < 0.05 between treatments determined by a one-way ANOVA with Dunnett’s multiple comparison test (**A**, **C**–**F**) or unpaired Student's *t*-test (*B*). Statistically significant differences are indicated by asterisks (* *P* < 0.05; ** *P* < 0.01; *** *P* < 0.001; **** *P* < 0.0001)

Two non-overlapping regions of the *Dg-CatD-1* gene were selected for synthesis of dsRNA. When these dsRNA regions were fed to adult female mites, at a final concentration of 200 ng/ μl in a blood meal, both resulted in significant levels of *Dg-CatD-1* knockdown relative to control (*lacZ*) mites (one-way ANOVA, *F*_(2,9)_ = 5.342, *P* = 0.0296) (Fig. 2A). Both regions achieved similar levels of knockdown—61% (± 23%, *P* = 0.0185) knockdown for region 1 and 64% (± 22%, *P* = 0.0149) knockdown for region 2—relative to the negative control (*lacZ*-fed) mites (Fig. 2A). When the two non-overlapping regions of *Dg-CatD-1* dsRNA were mixed and in equimolar amounts when fed to adult female *D. gallinae*, at a final combined concentration of 200 ng/μl, an increase in *Dg-CatD-1* knockdown was observed, with *Dg-CatD-1* levels reduced by 91% (± 7%) compared with control mites exposed to *lacZ* dsRNA (unpaired Student’s *t*-test, *t*_(6)_ = 13.82, and *P* < 0.0001) (Fig. 2B).

#### *Dg-CatD-1* dsRNA concentration optimisation experiments

To determine the optimal dsRNA concentration required for efficient gene silencing, *Dg-CatD-1* dsRNA (for region 2, 485 bp) was delivered at concentrations ranging from 400 ng/μl to 12.5 ng/μl to adult female mites in a blood meal (Fig. 2C and D). In the first and second experiments (Fig. 2C and D, respectively), knockdown of *Dg-CatD-1* followed a dose response, with higher concentrations of dsRNA more effective at gene silencing relative to control (*lacZ*-fed) mites.

##### Concentration experiment 1

*Dg-CatD-1* dsRNAs for region 2 were fed to adult female mites in a blood meal at final concentrations of 400, 200, and 100 ng/μl (Fig. 2C). This affected *Dg-CatD-1* gene expression (one-way ANOVA, *F*_(3,15)_ = 5.048, *P* = 0.0129). Differences in mean *Dg-CatD-1* gene expression were statistically significant (Dunnett’s multiple comparisons) between *Dg-CatD-1* dsRNA-fed and the *lacZ* control group at 400 ng/μl (79% knockdown, *P* = 0.0058) and 200 ng/μl (61% knockdown, *P* = 0.0301) (Fig. 2C).

##### Concentration experiment 2

*Dg-CatD-1* dsRNAs for region 2 were fed to adult female mites in a blood meal at final concentrations of 200, 100, 25, and 12.5 ng/μl (Fig. 2D). This affected *Dg-CatD-1* gene expression (one-way ANOVA, *F*_(4,18)_ = 8.500, *P* = 0.0005). Differences in mean *Dg-CatD-1* gene expression were statistically significant (Dunnett’s multiple comparisons) between the *Dg-CatD-1* dsRNA-fed and the *lacZ*-fed control group at 200 ng/μl (76% knockdown, *P* = 0.0004), 100 ng/μl (56% knockdown, *P* = 0.0064), and 25 ng/μl (42% knockdown, *P* = 0.0398) (Fig. 2D).

#### *Dg-CatD-1* dsRNA length optimisation experiments

To determine the optimal and minimum length of dsRNA required for efficient gene silencing, a series of *Dg-CatD-1* dsRNAs were generated with fragment length ranging from 500 bp to 25 bp (Fig. 2E and F).

##### Length experiment 1

*Dg-CatD-1* dsRNAs of 500, 300, 200, and 150 bp were fed to adult female mites (at a final concentration of 200 ng/μl) in a blood meal (Fig. 2E). This affected *Dg-CatD-1* gene expression (one-way ANOVA, *F*_(4,19)_ = 5.722, *P* = 0.0034). All tested *Dg-CatD-1* dsRNAs achieved similar levels of knockdown (Dunnett’s multiple comparisons) relative to control (*lacZ*) mites: 500 bp, 74% knockdown (*P* = 0.0003); 300 bp, 61% knockdown (*P* = 0.0020); 200 bp, 62% knockdown (*P* = 0.0018); 150 bp, 62% knockdown (*P* = 0.0020) (Fig. 2E).

##### Length experiment 2

The length of *Dg-CatD-1* dsRNA tested was further reduced. *Dg-CatD-1* dsRNAs of 200, 100, 50, and 25 bp were fed to adult female mites (at a final concentration of 200 ng/μl) in a blood meal (Fig. 2F). This affected *Dg-CatD-1* gene expression (one-way ANOVA, *F*_(4,20)_ = 7.778, *P* = 0.0006). All tested *Dg-CatD-1* dsRNAs again achieved similar levels of knockdown [Dunnett’s multiple comparisons relative to control (*lacZ*-fed) mites: 200 bp, 86% knockdown (*P* = 0.0034); 100 bp, 72% knockdown (*P* = 0.0080); 50 bp, 84% knockdown (*P* = 0.0023); 25 bp, 61% knockdown (*P* = 0.0251) (Fig. 2F]. In conclusion, a wide range of *Dg-CatD-1* dsRNA lengths (25–500 bp) efficiently knock down *Dg-CatD-1* expression levels.

### Phenotypic analysis of* Dg-CatD-1* silencing

**Fig. 3:**
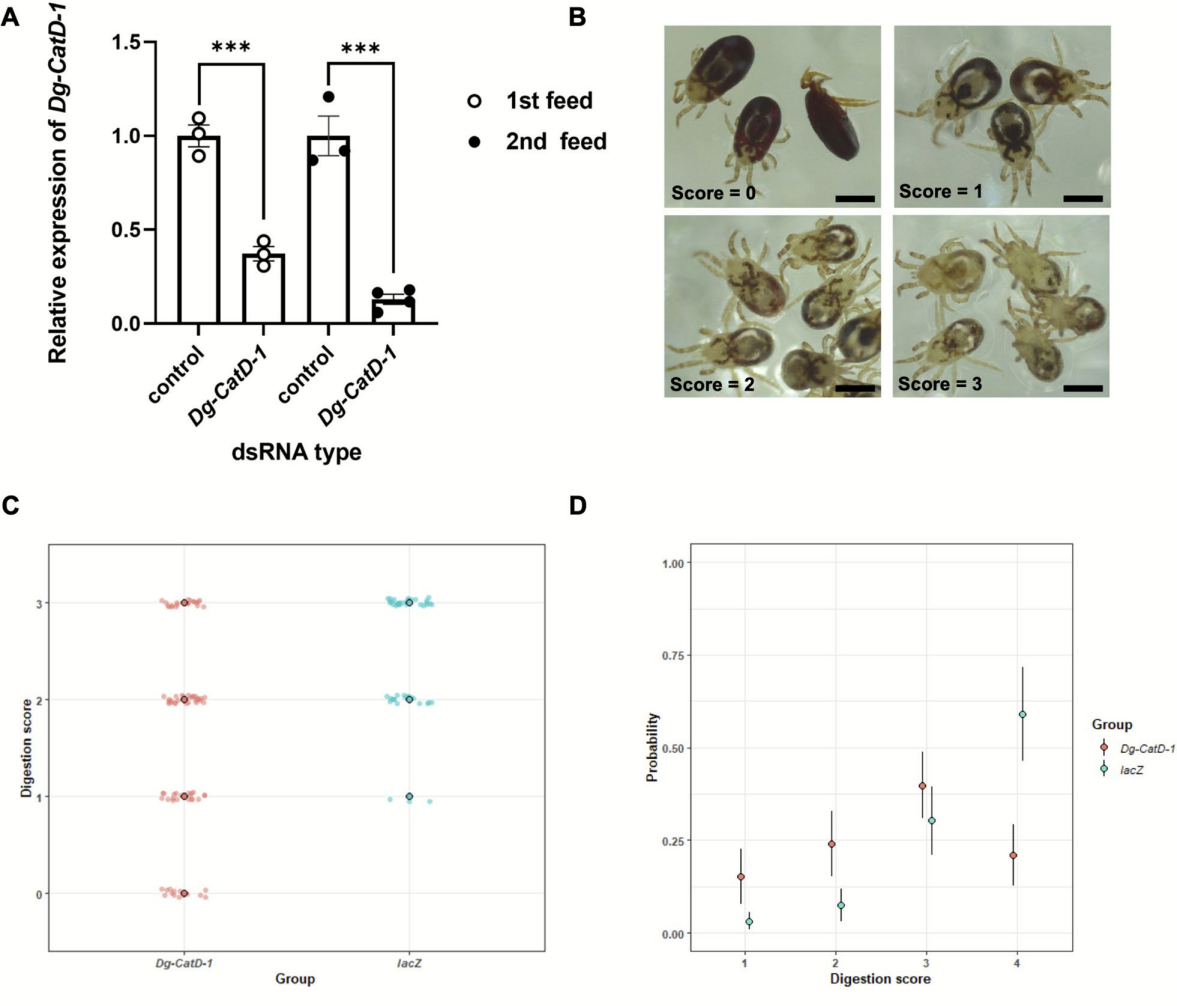
RNAi-mediated knockdown of *Dg-CatD-1* impacts blood meal digestion in adult female mites. **A** Real-time RT-qPCR analysis of *Dg-CatD-1* expression in adult female *D. gallinae* following one (first feed) or two (second feed) rounds of *Dg-CatD-1* dsRNA or control (*lacZ*) dsRNA feeding. Each dsRNA was delivered at a final concentration of 200 ng/μl in goose blood. Expression of *Dg-CatD-1* was monitored at 72 h post-dsRNA delivery. Mites receiving one round of dsRNA feeding are shown as empty circles, and those receiving a second round of dsRNA are shown as solid circles. Expression of *Dg-CatD-1* was normalised to the average of three *D. gallinae* housekeeping genes (*Dg-GAPDH*, *Dg-apoLp-1*, and *Dg-EF-1α-1*), and expression of *Dg-CatD-1* is shown relative to control (*lacZ*-fed) mites. Individual data points for biological replicates are shown with mean ± standard error of the mean (SEM) (*n* = 3). Asterisks represent significant difference at *P* < 0.05 between treatments as determined by unpaired Student *t*-tests (*** *P* < 0.001). **B** Overview of blood meal digestion score in adult female *D. gallinae*. Digestion scores from 0 to 3 were based on the amount of blood meal remaining in the mite body at 72 h post-feeding. A score of 0 represents an undigested blood meal; scores of 1 and 2 represent partially digested blood meals; a score of 3 represents fully digested blood meal. Scale bar = 500 μm. **C**, **D** Digestion scores of 0, 1, 2, and 3 were recorded at 72 h post-feeding following the second round of dsRNA feeding. **C** Each point represents the score of an individual *D. gallinae* mite after treatment with two rounds of gene-specific dsRNA. Scores were treated as a numeric value and presented with mean ± 95% confidence interval. **D** Digestion score was the response variable using ordinal models, and we fitted group as a fixed effect and replicate as a random effect. The probability is presented with mean ± standard error of fit for prediction

Following single-exposure feeding of dsRNA representing *Dg-CatD-1*, no increases in mite mortality or decreases in mite egg-laying were observed at any dsRNA concentration or length. Given the discrepancy between the likely importance of this gene to *D. gallinae* due to its blood-feeding behaviour and the lack of phenotype generated by substantial knockdown of its expression, a further in vitro mite feeding trial was conducted. Adult female *D. gallinae* were fed blood containing either *lacZ* dsRNA or *Dg-CatD-1* (region 1) dsRNA twice in sequential feeds with a gap of 72 h between feeds. As shown in Fig. 3A, RT-qPCR revealed effective gene silencing of *Dg-CatD-1* expression following both rounds of feeding compared to the *lacZ* control. Statistically significant differences in *Dg-CatD-1* expression were observed: For the “first feed” samples, an average reduction of 63% (± 7%) was shown (unpaired Student’s *t*-test, *t*_(4)_ = 9.007, *P* = 0.0008). For the “second feed” group, knockdown was also achieved, with an 87% (± 9%) reduction in gene expression (unpaired Student’s *t*-test *t*_(5)_ = 9.299, *P* = 0.0002).

Blood meal digestion in adult female *D. gallinae* was assessed to determine whether a second sequential dsRNA treatment would result in gene expression being reduced long enough to result in lower levels of Dg-CatD-1 protein, which may then affect the mite’s ability to digest a blood meal. This was scored based on blood digestion 72 h after only the second feeding of gene-specific dsRNA. A scoring system was applied in this study based on that developed previously [23]. As the blood meal was visible through the transparent cuticles of *D. gallinae*, a blood meal digestion score was given to each mite based on their dorsal morphological characteristics. Each score was based on the colour and volume of visible blood residues observed in the midgut, hindgut, and three parts of the caeca. A group of representative *D. gallinae* from this experiment with phenotypes corresponding to each digestion score (0–3) is shown in Fig. 3B. A significant difference was found in the digestion scores of *D. gallinae* fed on *Dg-CatD-1* or *lacZ* dsRNA for a second time. Our ordinal model showed that the *lacZ* group had a significantly higher digestion score than group 1 (estimate = 1.6891, SE = 0.3538, LRT = 11.28, *P* = 0.0007), with model estimates suggesting that *D. gallinae* in the *lacZ* group were more likely to have higher digestion scores, corresponding to the control mites having digested more of their blood meal than the “second-fed” *Dg-CatD-1* dsRNA mites (Fig. 3C, D).

### The impact of RNAi-mediated *Dg-CatD-1* gene knockdown on protein expression and haemoglobin metabolism

**Fig. 4:**
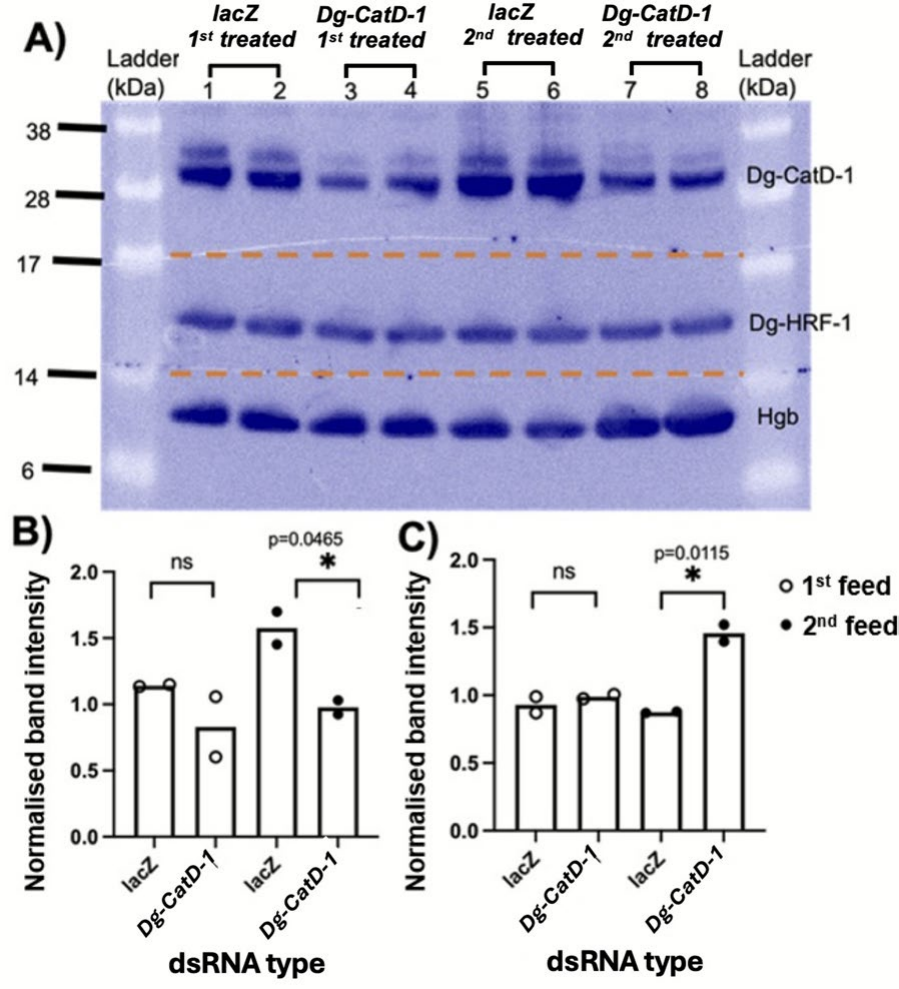
Densitometry analysis and gel band quantification of Dg-CatD-1, Dg-HRF-1, and haemoglobin after the first- and second-round feeding of *Dg-CatD-1* gene-specific dsRNA.** A** Western blot of soluble extracts of *D. gallinae* adult female mites immunostained for Dg-CatD-1, Dg-HRF-1, and avian haemoglobin (Hgb) proteins. The mites had been fed goose blood containing *lacZ* dsRNA for a single feed (lanes 1 and 2), *Dg-CatD-1* dsRNA for a single feed (lanes 3 and 4), *lacZ* dsRNA in two consecutive feeds (lanes 5 and 6), or *Dg-CatD-1* dsRNA for two consecutive feeds (lanes 7 and 8). The molecular weight markers (ladder) are indicated on the left. The nitrocellulose membrane was cut horizontally as indicated by the red dashed line at 14 kDa or 17 kDa prior to probing with the relevant antibody. The upper section of the membrane was probed with an anti-Dg-CatD-1 antibody raised in rabbit, the middle section was probed with an anti-Dg-HRF-1 antibody raised in rabbit, and the bottom section was probed with an anti-avian Hgb antibody raised in rabbit. Panels **B** and **C** illustrate the band density for Dg-CatD-1 (**B**) and Hgb (**C**); band densities of Dg-CatD-1 and Hgb were normalised to the Dg-HRF-1 band within the same lane. All data are shown in the mean, with individual values as points. Statistically significant differences of *P* < 0.05 by an unpaired Student *t*-test are indicated by asterisks (* *P* < 0.05)

In addition to the transcriptional and macroscopic impacts of RNAi-mediated *Dg-CatD-1* gene silencing, the impacts on Dg-CatD-1 protein and its haemoglobin substrate abundance were also determined through Western blot and densitometry analysis, as shown in Fig. 4A–C. The Dg-CatD-1 protein level was depleted in the *Dg-CatD-1* dsRNA-treated mites following both one and two rounds of gene silencing, with 27% and 38% lower quantities of the protein detected, respectively, than in *lacZ* dsRNA-treated mites. The *Dg-CatD-1* protein levels were statistically significantly impacted, however, only after two rounds of feeding on *Dg-CatD-1* dsRNA (Fig. 4B). Accordingly, avian haemoglobin band densities were similar between the *lacZ* and *Dg-CatD-1* dsRNA-treated mite extracts following the first feed, but following the second feed, the haemoglobin band density was significantly higher in the *Dg-CatD-1* dsRNA-treated mites than in *lacZ*-treated mites, indicating inhibition of haemoglobin digestion following this second round of RNAi (Fig. 4C).

### Impact of RNAi-mediated *Dg-CatD-1* gene knockdown on *D. gallinae* reproductive rates

**Fig. 5:**
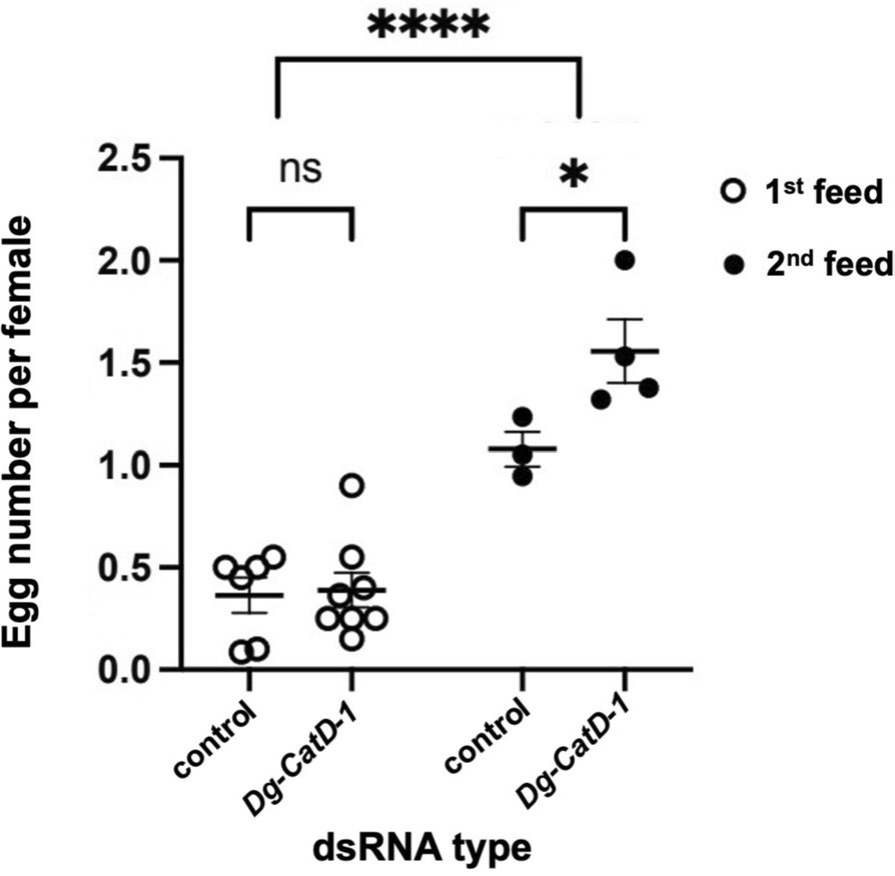
The impact on *D. gallinae* fecundity after RNAi-mediated *Dg-CatD-1* gene knockdown. Adult female *D. gallinae* mites were fed goose blood containing gene-specific dsRNA (*Dg-CatD-1*) or control dsRNA (*lacZ*) for one (first feed) or two consecutive feeds (second feed), 72 h apart. Each dsRNA was delivered at a final concentration of 200 ng/μl, and mites were then sorted into groups of 20 mites each for the monitoring of egg-laying rates. Depending on the different feeding rates, between three and eight groups were collected for this experiment (*n* = 3–8). Each point represents the average number of eggs laid by the females in that group by 72 h post-feeding. Mites receiving one round of dsRNA feeding are shown as empty circles, and those receiving a second round of dsRNA are shown as solid circles. All data are presented as mean ± SEM. Statistically significant differences of *P* < 0.05 calculated by a two-way ANOVA with Šidák post hoc multiple test correction are indicated by asterisks (* *P* < 0.05 and **** *P* < 0.0001)

Egg-laying rates were calculated as the average number of eggs laid per female in each treatment group (Fig. 5). Significant differences were observed between the first feed and the second feed groups (two-way ANOVA, *F*_(1,17)_ = 71.73, *P* < 0.0001), indicating that repeated feeding within the short experimental period increased oviposition. In the second feed group, *Dg-CatD-1* dsRNA-treated females produced more eggs than the control group (two-way ANOVA, *F*_(1,17)_ = 5.141, *P* = 0.0341). Eggs from all groups hatched within 1 week, with no differences in hatching rates by different treatments (Fig. 5).

## Discussion

In this study, we successfully used RNAi-mediated gene silencing to knock down the expression of *Dg-CatD-1*, an aspartyl proteinase from *D. gallinae*. Significant knockdown of *Dg-CatD-1* was achieved when dsRNA for one or both regions of the gene were delivered orally to *D. gallinae* in a blood meal. However, to observe an impaired blood digestion phenotype, we needed to give adult female *D. gallinae* two successive feeds with gene-specific dsRNA. This implies that sufficient Dg-CatD-1 enzyme was present in the lysosomes of mites before any dsRNA feeding to successfully digest the ingested haemoglobin after the first *Dg-CatD-1* dsRNA delivery. Gene silencing after consumption of two meals containing *Dg-CatD-1* dsRNA was then sufficient to prevent replenishment of the lysosomes with the quantities of the enzyme required for effective proteolysis of the haemoglobin in the blood meal. Although the reduction in haemoglobin digestion after two rounds of dsRNA feeding was moderate, these results demonstrate a clear functional impact. This outcome is consistent with our genomic analysis of the cathepsin-D family in *D. gallinae*, which reveals extensive gene duplication, with nine encoded copies showing substantial sequence divergence among paralogs. Importantly, sequence analyses indicate that six of these cathepsin-D family members are likely to encode functional enzymes based on conservation of active-site aspartic acid residues and may play a role in haemoglobin digestion. It remains to be determined whether RNAi knockdown using dsRNAs targeting each paralog will produce a more pronounced haemoglobin digestion phenotype.

Although *Dg-CatD-1* expression is inducible by blood-feeding [8], the transcript for this enzyme is present at high levels (3000–10,000 read counts even in starved haematophagous stages of the mite [4]), underpinning the argument that these high levels of constitutive expression likely result in abundant protein being present before the first gene-silencing event. Indeed, protein levels in organisms often remain stable despite significant fluctuations in mRNA abundance [27–29]. This stability is partly due to differences in protein turnover rates—some proteins degrade slowly and persist even after mRNA levels are reduced [27, 30]. Whilst protein levels generally correlate with mRNA abundance, translation efficiency and degradation rates also play major roles in determining protein expression [31]. Furthermore, regulation at the protein level helps to buffer against unwanted mRNA fluctuations, maintaining consistent protein concentrations [31]. This could explain why many published studies demonstrate substantial knockdown of critical genes yet fail to generate a phenotype. In *Arabidopsis thaliana,* no phenotype was seen despite the successful RNAi-induced downregulation of six target genes simultaneously, resulting in an 80% decrease in transcript levels [32]. Similarly, in the pea aphid *Acyrthosiphon pisum*, gene expression of *Ap-crt* and *Ap-cath-L* was reduced by 40% via dsRNA microinjection, without generating any phenotype [33]. Even in *D. gallinae,* when the essential gene *Dg-vATPase A* was significantly knocked down, there was no obvious phenotype [34], contrary to the dark-body phenotype induced by RNAi of this gene in the two-spotted spider mite, *Tetranychus urticae* [35].

RNAi-induced knockdown of *Dg-CatD-1* was successfully achieved through the oral delivery of dsRNA mixed with goose blood at concentrations ranging from 25 to 400 ng/μl (w/v). Higher concentrations of dsRNA resulted in increased knockdown, suggesting a dose–response relationship between the level of *Dg-CatD-1* gene silencing and the dsRNA concentration. At 12.5 ng/μl, knockdown levels were comparable to those observed in the *lacZ* control group, indicating that very low concentrations of dsRNA were insufficient to initiate effective silencing. This concentration-dependent effect on RNAi efficiency has been documented in other arthropod species [36–38]. In these studies, regardless of whether dsRNA was delivered via injection [38, 39] or orally [38, 40], higher concentrations consistently led to greater knockdown, supporting the findings observed in *D. gallinae*. It is also worth noting that dsRNA degradation has been reported following oral delivery of dsRNA in insect species [38, 41]. Delivering dsRNA at higher concentrations may help overcome this degradation and ensure adequate dsRNA uptake by the cells, enabling an effective RNAi response despite digestive breakdown within the mite.

Knockdown of *Dg-CatD-1* was also achieved when dsRNAs ranging from 25 to 500 bp were delivered at a fixed concentration of 200 ng/μl (w/v). The effect of dsRNA length on RNAi efficiency varies among different organisms and a universal minimum length for effective silencing has not been established. In *Diabrotica virgifera* (western corn rootworm), a minimum dsRNA length of 60 bp was required to induce RNAi [42], and in *T. urticae,* longer dsRNAs were found to be more effective in silencing the *TuCOPB2* gene and generating a spotless phenotype [43]. Similarly, in the red flour beetle (*Tribolium castaneum*), dsRNA lengths of 69 bp efficiently induced knockdown of enhanced green fluorescent protein (EGFP), while a shorter 31-bp dsRNA failed to knock down EGFP expression [39]. In *D. gallinae*, shorter dsRNA was capable of inducing knockdown of *Dg-CatD-1*, but these shorter sequences exhibited greater variability and yielded less significant differences in gene expression compared to longer dsRNAs. These results suggest that dsRNAs between 100 and 500 bp in length may offer the most reliable and efficient RNAi knockdown in *D. gallinae*, especially when delivered at a concentration of 200 ng/μl (w/v).

In this study, the effects of dsRNA concentrations and lengths were tested specifically for *Dg-CatD-1* in *D. gallinae*. Future research will explore whether these effects apply for other genes. Additionally, in the second concentration optimisation trial described here, statistically significant knockdown was successfully achieved at a dsRNA concentration of 100 ng/μl (w/v), whereas the knockdown observed in the first trial at this concentration was not statistically significant. The lack of significance in the first trial may be attributed to high variability in *Dg-CatD-1* expression within the *lacZ* negative control group, as shown in Fig. 2A. As discussed above, the expression of *Dg-CatD-1* is inducible by blood-feeding in *D. gallinae* [8], and so could vary depending on the amount of blood individual mites have consumed, leading to variability in the control group.

Sampling time post-dsRNA delivery in a blood meal in this study was based on previous RNAi studies, where it was demonstrated that the expression of *Dg-vATPase A* was significantly reduced after 24 h of RNAi induction and was persistently knocked down for at least 120 h [34] Also, due to the selection of genes heavily involved in the haemolytic and proteolytic cascades in the work presented here, sampling mites at 72 h post-dsRNA delivery was the optimal time frame to allow *D. gallinae* to carry out blood meal digestion and oviposition, as well as allowing RNAi to have a functional impact.

Using optimised RNAi methodology, we demonstrated that silencing of the transcript representing *Dg-CatD-1* resulted in a reduction of the abundance of this enzyme and inhibition of haemoglobin digestion, as assessed by both the presence of undigested blood in the whole mites and a considerable increase in the quantity of haemoglobin detected in mite lysate. Aspartyl proteinases initiate haemoglobin digestion in a range of parasites, including the malarial parasite, *Plasmodium falciparum* [44], the castor bean tick, *Ixodes ricinus* [14], the blood fluke, *Schistosoma mansoni* [45], and the New World hookworm, *Necator americanus* [16]. Inhibiting cathepsin D activity through proteinase inhibitors, vaccines, or RNAi has demonstrated the critical importance of this enzyme in haematophagous parasites and opened new avenues for parasite control. Vaccines based on cathepsin D have induced mortality in both endoparasites such as *Schistosoma japonicum* [46] and ectoparasites such as *D. gallinae* [7], as well as impacting reproduction in this latter species [6]. Following consumption of blood containing antibodies against Dg-CatD-1, three  phenotypes have previously been recorded: increased mortality [7] ; reduced fecundity [6] and reduced bloodmeal digestion [17]. Of these, only the latter phenotype was induced by gene silencing of *Dg-CatD-1* in this study, and only after a second dsRNA treatment. This suggests that RNAi may be useful as a screening tool for vaccine candidates in this species [47], but that not all the desirable impacts of a full immunological response to the antigen may be measurable by gene silencing.

Studies in other acarine species have reflected what has been demonstrated here for *D. gallinae*: RNAi of a cathepsin D in the tick *I. ricinus* resulted in gene-specific reductions in expression at the transcriptomic and protein level in the tick gut and a reduced ability to digest haemoglobin compared to the experimental control [12]. In the work presented herein, an increase was observed in the egg-laying rates of mites which had fed twice on blood following starvation in their conditioning period compared to mites which had fed only once after conditioning. This is in agreement with previous work where repeated feeding of previously starved *D. gallinae* increased their egg-laying rates [48]. Intriguingly, unlike the reduced fecundity observed in a tick species, *Haemaphysalis longicornis*, after RNAi of digestive enzyme [49], the fecundity rate was slightly increased by repeatedly feeding *Dg-CatD-1* dsRNA compared to controls in *D. gallinae*. Silencing of the spermathecal-related genes in *Aedes aegypti* also led to an increased fecundity rate [50]. One possible explanation is that exogenous dsRNA in the *D. gallinae* blood meal induced additional stress, triggering a stress response and resulting in higher egg-laying rates than the control. A similar phenomenon of increased egg-laying rates during stress has been observed in other mite species. For example, in the presence of predatory mites, *T. urticae* laid three to seven times the number of eggs as controls without predator exposure [51].

## Conclusions

RNAi can be used to identify and validate gene function in *D. gallinae*. For robust RNAi, at least for *Dg-CatD-1,* dsRNA should be at least 100 bp long and at concentrations of 200 ng/μl. RNAi-mediated gene knockdown of the aspartyl proteinase *Dg-CatD-1* effectively silenced gene expression levels in *D. gallinae*, and repeated delivery of dsRNA prolonged and enhanced these RNAi-mediated effects, resulting in reduced Dg-CatD-1 protein quantity and inhibited blood meal digestion. Finally, it is clear from this study that successful gene knockdown does not always result in a phenotype. However, following repeated bouts of gene knockdown, these phenotypic differences may become apparent. This does not necessarily reflect a lack of a biological impact, and therefore future studies relying on the use of RNAi for target gene selection should take into account the potential impact of residual protein levels, which may hide or cover any potential phenotypic effect.

## Supplementary Information

Additional file1 (XLSX 20 kb)

Additional file2 (DOCX 110 kb)

Additional file3 (DOCX 25 kb)

## Data Availability

All raw data and materials are available within the main text or supplementary information.

## Abbreviations

ANOVA: Analysis of variance
BLAST: Basic Local Alignment Search Tool
bp: Base pair
cDNA: Complementary DNA
*Dg-apoLp-1*: *Dermanyssus gallinae* apolipophorins-like
*Dg-CatD-1*: *Dermanyssus gallinae* cathepsin D-1
*Dg-eEF1a1-1*: *Dermanyssus gallinae* elongation factor 1-alpha 1-like
*Dg-GAPDH-1*: *Dermanyssus gallinae* glyceraldehyde 3-phosphate dehydrogenase-1
*Dg-HRF-1*: *Dermanyssus gallinae* histamine-releasing factor
dsDNA: Double-stranded DNA
dsRNA: Double-stranded RNA
EDTA: Ethylenediaminetetraacetic acid
Oligo: Oligonucleotide sequence
PCR: Polymerase chain reaction
RH: Relative humidity
RNAi: RNA interference
RT-qPCR: Reverse transcriptase quantitative polymerase chain reaction
SDS-PAGE: Sodium dodecyl sulfate–polyacrylamide gel electrophoresis
TAE: Tris-acetate–EDTA
TBST: Tris-buffered saline with Tween 20
