# Supplementary material for: RNAi-mediated knockdown of the poultry red mite cathepsin D-1 impacts haemoglobin digestion
Source: Parasit Vectors. 2026 Feb 6;19:109. doi: 10.1186/s13071-026-07254-y (PMC12973575; doi:10.1186/s13071-026-07254-y)
Supplement: Supplementary file 2 — Additional file2 (DOCX 110 kb) [file 13071_2026_7254_MOESM2_ESM.docx]

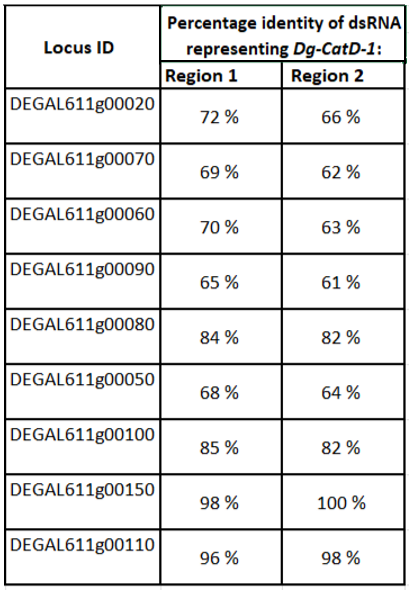


**Table S2.** Percentage nucleotide identity between Region 1 and Region 2 of *Dg-CatD-1* (HE565350) and *Dg-CatD* orthologs present in the *D. gallinae* genome.
