# Supplementary material for: RNAi-mediated knockdown of the poultry red mite cathepsin D-1 impacts haemoglobin digestion
Source: Parasit Vectors. 2026 Feb 6;19:109. doi: 10.1186/s13071-026-07254-y (PMC12973575; doi:10.1186/s13071-026-07254-y)
Supplement: Supplementary file 3 — Additional file3 (DOCX 25 kb) [file 13071_2026_7254_MOESM3_ESM.docx]

CLUSTAL O(1.2.4) multiple sequence alignment CDS

DEGAL611g00090 ------------------------------------------------------------ 0

DEGAL611g00050 ATGGTCACCGCGAAGCAGCATGTGTTGGTGACGGCGACGGGCCTCGCCACGATCCTCATT 60

DEGAL611g00070 ------------------------------------------------------------ 0

DEGAL611g00060 ------------------------------------------------------------ 0

DEGAL611g00020 ATGAAGTCATCGCGGT------CGCTATCGATGGCG------ACGCGCGTCGTCCTCGTA 48

DEGAL611g00100 ATGAAGCGTCAGCGGATGCCGACGATAATGATGGCGGCGGCTATGGCGGTTATCGTGGCC 60

DEGAL611g00080 ------------------------------------------------------------ 0

DEGAL611g00110 ------------ATGAAGGGTCAGCGGATGATGATGGCGGCTATGCTTGTCATCGTGGCC 48

HE565350.1 ---------------------------------ATGGCGGCTATGGTCGCCATCGCGGCC 27

DEGAL611g00150 ------------------------------------------------------------ 0

DEGAL611g00090 ------------------------------------------------------------ 0

DEGAL611g00050 TGGTTGATGTGCACGTCATGCACAAAC---------------GCCGACCTCATCAGGATG 105

DEGAL611g00070 ------------------------------------------------------------ 0

DEGAL611g00060 ------------------------------------------------------------ 0

DEGAL611g00020 CTGTGGGCGGCGTCATGTGCCGCCCAAGCCGGCCTCATGAGGGTACCCCTGCTCAAGATG 108

DEGAL611g00100 CTGTGCA---CGGGGCCGCGCGCCGGCGCCGATCTCATCAGGGTGCCTCTGAAGAAGATG 117

DEGAL611g00080 ---------------------------------------------------------ATG 3

DEGAL611g00110 CTCTG------CACGGCGTGCGCCGGCGCCGATCTCATCAGG------------------ 84

HE565350.1 CTGTG------CACGGCGTGCGCCGGCGCCGATCTCATCAGGGTGCCTCTGAAAAAGATG 81

DEGAL611g00150 ------------------------------------------------------------ 0

DEGAL611g00090 ------------------------------------------------------------ 0

DEGAL611g00050 GAACGTCCTCGGGCTCGAACGTTAACTCACCGCGTGCCGTTGAGTCTCA----------- 154

DEGAL611g00070 ------------------------------------------------------------ 0

DEGAL611g00060 ------------------------------------------------------------ 0

DEGAL611g00020 GAGACCATTCGGTCGCAAATGATGTCGAAAAATACACCGCGTCAATTGTTGCACTCACAG 168

DEGAL611g00100 GAGAGTGCTCGCGCTCACATGGTACCGCAGGAAGTATCGCTAAATGCCA----------- 166

DEGAL611g00080 GAGAGTGCTCGCGCTCACATGGTACCGCAGGAAGTATCGCTAAATGCCA----------- 52

DEGAL611g00110 ------------------------------------------------------------ 84

HE565350.1 GAGAGTGCTCACGCTCGCATGTTATCGCAGGACGTACCGCTGAATTTCA----------- 130

DEGAL611g00150 ------------------------------------------------------------ 0

DEGAL611g00090 ---------------------------------------------ATGTGGTTACAGGTC 15

DEGAL611g00050 -AAAACAACCGGTATCCAACTAGAAACGGCACTGACCCACTTCGCAACAACAGGGACGTC 213

DEGAL611g00070 ---------------------------------ATGCACAAACGCCGACCTCATCAGGTA 27

DEGAL611g00060 ---------------------------------------------ATGCGGTTACAGCTA 15

DEGAL611g00020 TCAGCAGGCGTCAATGGAGTCAAAGGAAGCGTTGAGCCTATCAACAACTATATGGATGCA 228

DEGAL611g00100 -TGTTCAACCAGCTTCGACCTAAGAAAGGCATTATACCTCTTTACAACTTCAATGACGTG 225

DEGAL611g00080 -TGTTCAACCAGCTTCGACCTAAGAAAGGCATTGTACCTCTTAACAACTTCAATGA---- 107

DEGAL611g00110 ------------------------------------------------GTGCCTCTGAAG 96

HE565350.1 -TCTTCAACCAGCTTCGACCCAAAAAGGGCATTGAGCCCCTTAACAACTTCGGAGACGCG 189

DEGAL611g00150 ------------------------------------------------------------ 0

DEGAL611g00090 GAATACTACGGCCCAATCACAATCGGGACCCCACCGCAGACGTTCCAGGTAATCTTCGAC 75

DEGAL611g00050 GAATACTACGGCCCAATCACAATCGGGACCCCACCGCAGACGTTCCAGGTAATCTTCGAC 273

DEGAL611g00070 GAATACTACGGCCCAATAACAATCGGGACCCCGCCGCAGACTTTCCAAGTAGTCTTCGAC 87

DEGAL611g00060 GAATACTACGGCCCAATAACAATCGGGACCCCGCCGCAGACGTTCCAAGTAGTCTTCGAC 75

DEGAL611g00020 CAATACTACGGCCCGATCTCAATCGGCAGCCCGCCACAGCCATTCCAGGTCGTTTTCGAT 288

DEGAL611g00100 CAATATTACGGCCCGATCACAATTGGCACACCACCGCAGACGTTTCAGGTAATCTTTGAT 285

DEGAL611g00080 -----------------------------------------------CGTAATCTTTGAT 120

DEGAL611g00110 AAGATGGAGAGTGCTCACGCTCGCATGTTATCGCAGGACACGTTCCAGGTGGTCTTTGAT 156

HE565350.1 CAATACTACGGCCCGATCACAATCGGAACGCCACCGCAGACGTTCCAGGTGATCTTTGAT 249

DEGAL611g00150 ------------------------------------ATGAAGGGTCAG-----------C 13

DEGAL611g00090 ACTGGATCATCGGATCTCTGGATACCCTCGTCCAAGTG---------------------- 113

DEGAL611g00050 ACTGGATCATCGGATCTCTGGATACCCTCGTCCAAGTGCCACGACGCG------GTTTGC 327

DEGAL611g00070 ACCGGATCATCGGAATTGTGGGTGCCCTCGTCCAAGTGCCACGAAGCG------CTCTGC 141

DEGAL611g00060 ACCGGATCATCGGATTTATGGGTACCCTCGTCCAAGTGCCACGAAGCG------CTGTGC 129

DEGAL611g00020 ACTGGCTCATCGGATCTTTGGGTGCCCTCTTCCAAATGTCCACTTACCAATATTGCATGC 348

DEGAL611g00100 ACCGCATCGTCGAATCTTTGGGTGCCTTCGTCCAAGTGCCCTAGCTCCAACGTTGCCTGC 345

DEGAL611g00080 ACCGCATCGTCGAATCTTTGGGTGCCTTCGTCCAAGTGCCCTAGCTCCAACGTTGCCTGC 180

DEGAL611g00110 ACCGGCTCGTCGGATCTTTGGGTGCCCTCGTCCAAGTGTCCCAGCTCTAACATTGCATGT 216

HE565350.1 ACCGGCTCGTCGGATCTTTGGGTGCCCTCGTCCAAGTGTCCCAGCTCTAACATTGCCTGT 309

DEGAL611g00150 GGATGATGATGGCGGCTATGGTCGCCATCGCGGCCC-TGTGCACGGCGTGCGCCGGCGCC 72

* * * * *** ** **

DEGAL611g00090 ------------------------------------------------------------ 113

DEGAL611g00050 GCACAACATAATAGATACGATGCCGGGAAGTCATCGACATACGTCGCGAATGGCCGATAC 387

DEGAL611g00070 GCAAAACGTAATAGATACGATGCCGAGAAGTCATCGACATACGTCGCGAATGGCAGAGAG 201

DEGAL611g00060 GCAAAACGTAATAGATACGATGCCGAGAAGTCATCGACATACGTCGCGAATGGCAGAGAG 189

DEGAL611g00020 CTGCTGCATAACAAGTACCATTCGGACAAATCGTCGACATACGTCAAAAACGGAACTGAA 408

DEGAL611g00100 AGGACGCACAATAAGTACGATGCGGAGAAGTCATCGACGCACGTCGCGAATGGCACCAAG 405

DEGAL611g00080 AGGACGCACAATAAGTACGATGCGGAGAAGTCATCGACGCACGTCGCGAATGGCACCAAG 240

DEGAL611g00110 GCGACGCACAGTAAGTACAATGCGGAGAAGTCATCGACGTACGTCGCAAATGGCACCAAG 276

HE565350.1 GCGACGCACAGTAAGTACAATGCGGAGAAGTCATCGACGTACGTCGCGAATGGCACCAAG 369

DEGAL611g00150 GATCTCATCAGTAAGTACAATGCGGAGAAGTCATCGACGTACGTCGCGAATGGCACCAAG 132

DEGAL611g00090 -----------------CCACGACGCGGTTTGCGGCTTTCTCTCAATGGATACGCTAAGC 156

DEGAL611g00050 TTCCTTATCACATATGGCTCGGGTGAAGTTGAAGGCTTTCTCTCAATGGATACGCTGAGC 447

DEGAL611g00070 TTCAATGTCACATATGGCTCGGGTGCAGTTCGAGGCTTTCTCTCAATGGATACGTTGAGC 261

DEGAL611g00060 TTCAATATCACATATGGCTCGGGTGCAGTTCGAGGCTTTCTCTCAATGGATACGTTGAGC 249

DEGAL611g00020 TTCAAAATTCAGTACGGCTCGGGCGCGGTGAGCGGCGTCCTGTCGGCGGACACGGTTGAT 468

DEGAL611g00100 TTTCAGTGTATTTACCCCTTGGGTTCCATATCGGGCGAGCTTTCAACGGACACGGTGAGA 465

DEGAL611g00080 TTTCAGTGTATTTACCCCTTGGGTTCCATATCGGGCGAGCTTTCAACGGACACGGTGAGA 300

DEGAL611g00110 TTCACGATTCAGTACGGCTCAGGATCCGTATCGGGCGTGCTTTCCACGGATACGGTGAGC 336

HE565350.1 TTCGCGATTCAGTACGGCTCAGGATCCGTATCGGGCGTGCTTTCCACGGATACGGTGAGC 429

DEGAL611g00150 TTCGCGATTCAGTACGGCTCAGGATCCGTATCGGGCGTGCTTTCCACGGATACGGTGAGC 192

* * * *** ** ** *** *** *

DEGAL611g00090 GTGAGTGGCCTTCGTGTGATGAACCAAACATTTGCCGAAGCTACATATGAGTCGGACG-- 214

DEGAL611g00050 GTGAGTGGCCTTCGTGTGATGAACCAAACATTTGCCGAAGCTACATCTGAGTCGAAAG-- 505

DEGAL611g00070 GTGAGTGGCATTCGTGTGATGAACCAAACATTCGCCGAAGCTACATGGGAGTCGGAAC-- 319

DEGAL611g00060 GTGAATGGCCTTCGTGTGACCAACCAAACATTCGCCGAAGCTACATGGGAGTCGGAAG-- 307

DEGAL611g00020 CTGAATGGTATGCGCGTCACCAACCAGACGTTTGCCGAGATCATGCGCGAATCGGGCCTC 528

DEGAL611g00100 GTGGGCGGCCTTACTGTCACCAAACAAACGTTTGCCGAGATCACGGAGGAGCCGGATG-- 523

DEGAL611g00080 GTGGGCGGCCTTACTGTCACCAAACAAACGTTTGCCGAGATCACGGAGGAGCCGGATG-- 358

DEGAL611g00110 GTGAGCGGCATTACGGTTACCAAGCAAACGTTTGGCGAGATCACGAAGGAGTCGGGTGAC 396

HE565350.1 GTGAGCGGCATTACGGTCACCAAGCAGACGTTTGGCGAGATCACAGAGGAGTCGGGTGAC 489

DEGAL611g00150 GTGAGCGGCATTACGGTCACCAAGCAGACGTTTGGCGAGATCACAGAGGAGTCGGGTGAC 252

** ** * ** * ** ** ** ** * *** * ** **

DEGAL611g00090 -ACTTCAACGCTGAACCTATTGACGGCATTCTCGGCATGGGCTATCCGGAACTCGCAACA 273

DEGAL611g00050 -ACTTCAGCGCTAACCCTATTGACGGCATTCTCGGCATGGGCTATCCGGAACTCGCAGAA 564

DEGAL611g00070 -ACTTCAGCGCTAAACCTATTGACGGCATTCTCGGCATGAGCTATCCGGACCTCGCAACA 378

DEGAL611g00060 -GCTTCAGCACTGACCCTATTGACGGCATTCTCGGCATGGGCTATCCACAACTTGCAAGA 366

DEGAL611g00020 GGCTTTATAGCTGGAAAGTTCGACGGCATCCTCGGTATGGGCTATCCGACGATCGCCA-- 586

DEGAL611g00100 -CCTTCGCCTACGGCAAATATGACGGCATTCTTGGCATGGGTTTTCCGGAGATCTCAA-- 580

DEGAL611g00080 -CCTTCGCCTACGGCAAATATGACGGCATTCTTGGCATGGGTTTTCCGGAGATCTCAA-- 415

DEGAL611g00110 TCCTTCATCTACGGAAAGTTTGACGGTATTCTTGGCATGGGCTATCCGGAAATCGCAA-- 454

HE565350.1 TCCTTCATCTACGGAAAGTATGACGGTATTCTTGGCATGGGCTATCCGGAAATCGCAA-- 547

DEGAL611g00150 TCCTTCATCTACGGAAAGTATGACGGTATTCTTGGCATGGGCTATCCGGAAATCGCAA-- 310

*** ***** ** ** ** *** * * *** * *

DEGAL611g00090 CCTGGAGCCCTGCCCGTCTTTGATCAGATGATGGCACAGAAAGTCATCGACAAGGCCGTG 333

DEGAL611g00050 TCTCGAGCCCTGCCCGTCTTTGATCAGATGATGGCACAGAAAGTCATCGACAAGGCCGTG 624

DEGAL611g00070 TCTGGAGCCCTGCCTGTCTTTGATCAGATGATGGCACAGAAAGTCATCGACAAGGCCGTG 438

DEGAL611g00060 TCTCGAGCCCTGCCCGTCTTTGATCAGATGATGGCACAGAAAGTCATCGACAAGGCCGTG 426

DEGAL611g00020 -GGGGTGGCCTACCGGTCTTCGACCAGATGGTGGCGCAGAACGTCATCGACCAGGCCGTC 645

DEGAL611g00100 -TCAGTGGCTTGCCCGTCTTCGACCAGATGGTGGAACAAAAAGTAGTCGAAAAGGCCATG 639

DEGAL611g00080 -TCAGTGGCTTGCCCGTCTTCGACCAGATGGTGGAACAAAAAGTAGTCGAAAAGGCCATG 474

DEGAL611g00110 -GCAGTGGCCTGCCCGTATTCGACCAGATGGTGAAACAAAAGGTCGTCGAAAAAGCCATC 513

HE565350.1 -GCAGCGGCCTGCCCGTTTTCGACCAGATGGTGAAACAAAAGGTCGTCGAAAAGGCCATC 606

DEGAL611g00150 -GCAGTGGCCTGCCCGTTTTCGACCAGATGGTGAAACAAAAGGTCGTCGAAAAGGCCATC 369

* * * * ** ** ** ** ****** ** ** ** ** **** * *** *

DEGAL611g00090 TTTTCCTTTTACCTCACCCGCGACCCTGAACACCTTCCTGGCAGCGAACTCGTTTTAGGA 393

DEGAL611g00050 TTCTCCTTTTACCTCACCCGCGACCCTAAACACCTTCCTGGCAGCGAACTCGTTTTAGGA 684

DEGAL611g00070 TTCTCCTTTTACCTCACCCGCGACCCTAAACACCTTCCTGGCAGCGAACTCGTTTTAGGA 498

DEGAL611g00060 TTTTCCTTTTACCTCACCCGCGACCCTAAACACCTTACTGGCAGCGAACTCGTTTTAGGA 486

DEGAL611g00020 TTTACCTTCTTCCTCACCCGCGACCCCAACCACCCCACAGGCAGCGAGCTCGTTTTAGGA 705

DEGAL611g00100 TTTTCCTTCTTCATCACCCGTGACCAACAGCACCCCACTGGCAGCGAGCTCGTCTTAGGT 699

DEGAL611g00080 TTTTCCTTCTTCATCACCCGTGACCAACAGCACCCCACTGGCAGCAAGCTCGTCTTAGGT 534

DEGAL611g00110 TTCTCCTTCTTCCTCACCCGTGACCCAGAGCACCCCATTGGCAGCCAGCTGGTCTTGGGA 573

HE565350.1 TTTTCCTTCTTCCTCACCCGTGACCCACAGCACCCCATTGGCAGCGAGCTGGTCTTGGGA 666

DEGAL611g00150 TTTTCCTTCTTCCTCACCCGTGACCCACAGCACCCCATTGGCAGCGAGCTGGTCTTGGGA 429

** **** * * ******* **** * **** ****** * ** ** ** **

DEGAL611g00090 GGTGTGGATCGAAACCACTACAAGGGCAAAATTACTTACGTTCCTGTTACTCAGAAAAAA 453

DEGAL611g00050 GGTGTGGATCGAAACCACTACAAGGGTAAAATTACGTACGTTCCTGTTACTAAGAAAAAA 744

DEGAL611g00070 GGTGTGGATCGAAACCACTACAAGGGCAAAATTACGTACGTTCCTGTTACTAAGAAAAAA 558

DEGAL611g00060 GGTGTGGATCGAAAACACTACAAGGGCAAAATTACTTACGTCCCTGTTACTACGAAACAA 546

DEGAL611g00020 GGCATCGACCCGAAGCACCACAAGGGAGAAATTACCTACACCCCGGTCACCCGCAAAGGC 765

DEGAL611g00100 GGCATCGATCCGAATCACTACAAGGGAGAGATTACCTACGCTCCTCTAAACGTCGAAGGC 759

DEGAL611g00080 GGCATCGATCCGAATCACTACAAGGGAGAGATTACCTACGCTCCTCTAAACGTCGAAGGC 594

DEGAL611g00110 GGCATCGACACGAAGCACTACAAGGGCGACATTACCTACGCTCCTCTAACCCGCGAAGCC 633

HE565350.1 GGCATCGACCCGAAGCACTACAAGGGCGACATTACCTACGCTCCTCTAACCCGCGAAAGC 726

DEGAL611g00150 GGCATCGACCCGAAGCACTACAAGGGCGACATTACCTACGCTCCTCTAACCCGCGAAAGC 489

** * ** ** *** ******* * ***** *** ** * * **

DEGAL611g00090 TGGTGGGAGATCAAAATGGACAAGCTGACCGTGGATGGTCAGACAAAACTCCATCTGTGC 513

DEGAL611g00050 TATTGGGAGATCAAAATGGACAAGCTGACCGTGGATGGCCAGACGAAACTCCACCTGTGC 804

DEGAL611g00070 TGGTGGGAGATCAAAATGGACAAGCTGACCGTGGATGGCCAGACGAAACTCCACCTGTGC 618

DEGAL611g00060 TGGTGGGAGGTCAAAATGGACAAGCTGACCGTGGATGGCCAGACGAAACTCCACCTGTGC 606

DEGAL611g00020 TACTGGCAGTTCGGCGTTGACAAGATTGCAGTGAGTGGACATTCAGATGA---GTTGTGT 822

DEGAL611g00100 TACTGGCGGTTCAGAGTGGACAAGATGACACTGGGTGGAAAGACAGCTCC---AGTATGC 816

DEGAL611g00080 TACTGGCGGTTCAGAGTGGACAAGATGACACTGGGTGGCAAGACAGCTCC---AGTATGC 651

DEGAL611g00110 TACTGGCAGTTCAGAGTGGATACGGTGACACTGAATGGCAAGACAGCTCC---AGTGTGC 690

HE565350.1 TACTGGCAGTTCAGAGTGGATAAGGTGACACTGAATGGCAAGGCAGCTCC---AGTGTGC 783

DEGAL611g00150 TACTGGCAGTTCAGAGTGGATAAGGTGACACTGAATGGCAAGGCAGCTCC---AGTGTGC 546

* *** * ** * ** * * * * ** *** * * * **

DEGAL611g00090 AAGGGCGGGTGCCGCGCAATCGTCGATTCAGGCACGTCGTTTATTGTCGGGCCCGTCGAA 573

DEGAL611g00050 AAGGGCGGGTGCCGCGCAATCGTCGATTCAGGCACGTCGTTTATTGTCGGGCCCGTCGAA 864

DEGAL611g00070 AAGGGCGGGTGCCGCGCAATCGTCGATTCAGGCACGTCGTTTATTGTCGGGCCCGTCGAA 678

DEGAL611g00060 AAGGGCGGGTGCCGCGCAATCGTCGATTCAGGCACGTCGTTTATTGTCGGGCCCTTCAAA 666

DEGAL611g00020 AAGGGCGGCTGCCAAGCTATCGCCGATACGGGCACGTCGCTTATCGCCGGCCCCACAAAG 882

DEGAL611g00100 CAGAATGGCTGTCACGGCCTCGTCAACACGGGTATGTAT--------------------- 855

DEGAL611g00080 CAGAATGGCTGTCACGGCCTCGTCAACACGGGTATGTAT--------------------- 690

DEGAL611g00110 CAGAAGGGCTGTGAGGGCATCGCCGACACGGGTACGTCACTCTTCGTTGGCCCCACCGCG 750

HE565350.1 CAGAAGGGCTGTGAGGGCATCGCCGACACGGGTACGTCACTCTTCGTTGGCCCCACCGCG 843

DEGAL611g00150 CAGAAGGGCTGTGAGGGCATCGCCGACACGGGTACGTCACTCTTCGTTGGCCCCACCGCG 606

** ** ** * *** * * * ** * **

DEGAL611g00090 GAGGCTAAGCTACTCATAAAGACCTTGGGC---GGCAAGCGAGAGAACGATACGTACACA 630

DEGAL611g00050 GAGGCTAAGCGGCTTATAAAGACCTTGGGC---GGCAAGCGAGAGAACGATACGTACACA 921

DEGAL611g00070 GAGGCCATGCTGCTCATAAAGACCTTGGGC---GGCAAGTCAGAGATGGGTGCGTTCACT 735

DEGAL611g00060 GAGGCTATGCTGCTCATAAAGACCTTGGGC---GGCAAGCCAGAGAACGATATGTTCACA 723

DEGAL611g00020 GAGGTCACGAAGCTGAACGAGCTCATCGGCGCCGCGCCATTCATCGGCGGCGAGTACATT 942

DEGAL611g00100 ---GTTGCAGCTCTTGCTGCCCAGCTCGGCGCCCAGGAGTCCGCTTCTGGACAGTACGTG 912

DEGAL611g00080 ---GTTGCAGCTCTTGCTGCCCAGCTCGGCGCCCAGGAGTCCGCTTCTGGACAGTACGTG 747

DEGAL611g00110 GATGTTGCGGCACTCGCTAGTCAGCTTGATGCCCAGGAGACCGCCCCTGGCCTGTACCTC 810

HE565350.1 GATGTTGCGGCACTCGCTAGTCAGCTTGATGCCCAGGAGACCGCCCCTGGCCTGTACCTC 903

DEGAL611g00150 GATGTTGCGGCACTCGCTAGTCAGCTTGATGCCCAGGAGACCGCCCCTGGCCTGTACCTC 666

* ** * * * ** *

DEGAL611g00090 GTGGACTGTGACCGGGCGTCAAGTCTGCCCAATGTTGAGTTCACAATCGCCGGCAAAACA 690

DEGAL611g00050 GTGGACTGTGACCGGGCGTCAAGTCTGCCCAATGTTGAGTTCACAATCGCCGGCAAAACA 981

DEGAL611g00070 GTGGACTGTGACCGGGCGTCAAGTCTGCCTAACGTTGAGTTCACAATCGCCGGCAAAACA 795

DEGAL611g00060 GTGGACTGTGACCGGGCGTCAAGTCTGCCCAATGTTGAGTTCACAATCGCCGGCAAAACG 783

DEGAL611g00020 GTTAACTGCAAAAATCTGCCCAACATGCCCAATATCGAGTTCACGATTTCAAACAGGACG 1002

DEGAL611g00100 GTCGACTGTGAGAAGGCGGGGAGCCTGCCCAACATCGAGTTAGCAATTGCCGGCAGGCTG 972

DEGAL611g00080 GTCGACTGTGAGAAGGCGGGGAGCCTGCCCAACATCGAGTTAGCAATTGCCGGCAGGCTG 807

DEGAL611g00110 GTCGACTGTGAGAAGGCGGGAGACCTGCCCAACATCGAGTTTACAATCGCCGGCAGGCCG 870

HE565350.1 GTCGACTGTGAGAAGGCGGGAGACCTGCCCAACATCGAGTTTACAATCGCCGGCAGGCCG 963

DEGAL611g00150 GTCGACTGTGAGAAGGCGGGAGACCTGCCCAACATCGA---------------------- 704

** **** * * **** ** * **

DEGAL611g00090 TTCGAGCTCACCTCCAGTGATTACATCCTAAGGTGGAACCTCACCGGTCACACAGCCTGT 750

DEGAL611g00050 TTCGAGCTCACCTCCAGTGATTACATCCTAAGGTGGAACCTCACCGGTCACACAGCCTGT 1041

DEGAL611g00070 TTCAAGCTCACCCCCAGTGATTACATCCTAAGGTCT------------------------ 831

DEGAL611g00060 TTCGAGCTCACCTCCAGTGATTACATTCTAAGGTGGAGCCTCACCGGTCGCACAGCCTGT 843

DEGAL611g00020 TTCGTTCTCACTCCAGACGAATACATCCTCAAGATGAGCCAGGGCAGCATGCCGGTGTGC 1062

DEGAL611g00100 TTCGAGTTTACTCACGCCGAGTACATTGTCAGGTTAAATCAAAGCAGCGAAACGCGTTGC 1032

DEGAL611g00080 TTCGAGTTTACTCACGCCGAGTACATTGTCAGGTTAAATCAAAGCAGCGAAACGCGTTGC 867

DEGAL611g00110 TTCGAGCTCACTCCCCTCGATTACGTCGTCAGGATAAAGCAAAGCGGCCAAACCTTCTGC 930

HE565350.1 TTCGAGCTCACTCCCCTCGATTACGTCGTCAGGTTAAAGCAAAGCGGCCAAACCTTCTGC 1023

DEGAL611g00150 --------------------------------------------CGGCCAAACCTTCTGC 720

DEGAL611g00090 GTTTTGGGATTCGCACCCTT---TGAGTCTTACCCGCCTCTGTGGATTCTGGGCGATGTC 807

DEGAL611g00050 GTTTTGGGCTTCGCACCGGA---TGATACT------ACTCAGTGGATTCTGGGCGATGTC 1092

DEGAL611g00070 ------------------------------TACCCGCCTCAGTGGATTCTGGGCGATGTC 861

DEGAL611g00060 GTTTTGGGCTTCTTACCGGA---TGGTACTAACCCGCCTCTGTGGATTCTGGGCGATGTC 900

DEGAL611g00020 CTATCAGGTTTCATCGGCCTTGATGTCCCGCGTGATCCCGTCTGGATCCTGGGCGACGTC 1122

DEGAL611g00100 TTATTGGCTTTCCGAGGCGTGGATATCCCAAACTTTCCGATATGGTCTCTGGGTGATATC 1092

DEGAL611g00080 TTATTGGCTTTCCGAGGCGTGGATATCCCAAACTTTCCGATATGGTCTCTGGGTGATATC 927

DEGAL611g00110 GTGTTGGCCTTCCAAGGCATGGATAACCCAGATGATCCCATCTGGATTCTGGGTGATATC 990

HE565350.1 GTGTTAGCCTTCCAAGGCATGGATATCCCAGATGATCCTATCTGGATTCTGGGTGATATC 1083

DEGAL611g00150 GTGTTAGCCTTCCAAGGCATGGATATCCCAGATGATCCTATCTGGATTCTGGGTGATATC 780

* *** ***** ** **

DEGAL611g00090 ATGATGGGCAAGTACTTCACCGTCTTCGATCGTGAAAACGACCGCGTTGTAGGCGTTGTT 867

DEGAL611g00050 ATGATGGGCAAGTACTTCACCGTCTTCGATCGTGAAAATGACCGCGTTGGTTTTGCCGAG 1152

DEGAL611g00070 ATGATGGGCAAGTACTTCACCGTCTTCGATCGTGAAAATGACCGCGTTGGTTTTGCCGAG 921

DEGAL611g00060 ATGATGGGCAAGTACTTCACCGTCTTCGATCGTGAAAATGACCGCGTTGGTTTTGCCGAG 960

DEGAL611g00020 TTCATCGGCCGATACTTTACGGTTTTCGACCGCCAAAATGATCAAGTTGGCTTCGCCGAC 1182

DEGAL611g00100 TTCATTGCAAAGTACTTCACCGTCTTCGATCGTGAAAACCATCGCATTGGCTTTGCCGAT 1152

DEGAL611g00080 TTCATTGCAAAGTACTTCACCGTCTTCGATCGTGAAAACCATCGCATTGGCTTTGCCGAT 987

DEGAL611g00110 TTCATTGGCAAATACTTCACAGTCTTCGATCGTGAAAACAATCGCGTTGGCTTTGCCGAT 1050

HE565350.1 TTCATTGGCAAATACTTCACAGTCTTCGATCGTGAAAACAATCGCGTAGGCTTTGCCGAT 1143

DEGAL611g00150 TTCATTGGCAAATACTTCACAGTCTTCGATCGTGAAAACAATCGCGTTGGCTTTGCCGAT 840

* ** * ***** ** ** ***** ** **** * * * * * *

DEGAL611g00090 GGTGATTCCTATGGCGTCTCCTAG 891

DEGAL611g00050 GCCGCCTAA--------------- 1161

DEGAL611g00070 GCCGCCTAA--------------- 930

DEGAL611g00060 GCCGCCTAA--------------- 969

DEGAL611g00020 GCAGCCTAG--------------- 1191

DEGAL611g00100 GCCGCATAA--------------- 1161

DEGAL611g00080 GCCGCATAA--------------- 996

DEGAL611g00110 GCCGTCTAA--------------- 1059

HE565350.1 GCCGCATAA--------------- 1152

DEGAL611g00150 GCCGTCTAA--------------- 849

* * *

#

# Percent Identity Matrix - created by Clustal2.1 CDS

#

#

1: DEGAL611g00090 100.00 92.18 89.82 90.18 63.56 64.07 62.64 63.67 66.67 63.02

2: DEGAL611g00050 92.18 100.00 91.13 91.90 59.67 62.50 63.50 62.37 65.77 63.04

3: DEGAL611g00070 89.82 91.13 100.00 93.46 64.18 64.67 63.84 64.69 66.45 63.59

4: DEGAL611g00060 90.18 91.90 93.46 100.00 63.76 64.43 63.74 64.06 66.25 63.19

5: DEGAL611g00020 63.56 59.67 64.18 63.76 100.00 63.45 63.05 65.62 66.93 65.14

6: DEGAL611g00100 64.07 62.50 64.67 64.43 63.45 100.00 99.50 78.68 83.20 78.71

7: DEGAL611g00080 62.64 63.50 63.84 63.74 63.05 99.50 100.00 81.61 83.03 78.91

8: DEGAL611g00110 63.67 62.37 64.69 64.06 65.62 78.68 81.61 100.00 93.26 91.28

9: HE565350.1 66.67 65.77 66.45 66.25 66.93 83.20 83.03 93.26 100.00 93.40

10: DEGAL611g00150 63.02 63.04 63.59 63.19 65.14 78.71 78.91 91.28 93.40 100.00

CLUSTAL O(1.2.4) multiple sequence alignment PROTIEN

DEGAL611g00070 ------------------------------------------------------------ 0

DEGAL611g00060 ------------------------------------------------------------ 0

DEGAL611g00090 ------------------------------------------------------------ 0

DEGAL611g00050 MVTAKQHVLVTATGLATILIWLMCTS-CTNADLI-----RMERPRARTLTHRVPLSLKNN 54

DEGAL611g00020 MKSSRS---LSM--ATRVVLVLWAASCAAQAGLMRVPLLKMETIRSQMMSKNTPRQLLHS 55

DEGAL611g00100 MKRQRMPTIMMA--AAMAVIVALCTGPRAGADLIRVPLKKMESARAHMVPQEVSLNAMFN 58

DEGAL611g00080 ----------------------------------------MESARAHMVPQEVSLNAMFN 20

HE565350.1 -----------------------------GDAQYY-------G----------------- 7

DEGAL611g00110 MKGQ----RMMM--AAMLVIVALCTA-CAGADLIRVPLKKMES----------------- 36

DEGAL611g00150 MKGQ----RMMM--AAMVAIAALCTA-CAGADLI-------------------------- 27

DEGAL611g00070 ------------MHKRRPHQVEYYGPITIGTPPQTFQVVFDTGSSELWVPSSKCHE--AL 46

DEGAL611g00060 ----------------MRLQLEYYGPITIGTPPQTFQVVFDTGSSDLWVPSSKCHE--AL 42

DEGAL611g00090 ----------------MWLQVEYYGPITIGTPPQTFQVIFDTGSSDLWIPSSKCHD--AV 42

DEGAL611g00050 RY----PTRNGTDPLRNNRDVEYYGPITIGTPPQTFQVIFDTGSSDLWIPSSKCHD--AV 108

DEGAL611g00020 QSAGVNGVKGSVEPINNYMDAQYYGPISIGSPPQPFQVVFDTGSSDLWVPSSKCPLTNIA 115

DEGAL611g00100 QL----RPKKGIIPLYNFNDVQYYGPITIGTPPQTFQVIFDTASSNLWVPSSKCPSSNVA 114

DEGAL611g00080 QL----RPKKGIVPLNNFNDV-----------------IFDTASSNLWVPSSKCPSSNVA 59

HE565350.1 -------------------------PITIGTPPQTFQVIFDTGSSDLWVPSSKCPSSNIA 42

DEGAL611g00110 -------------------------AHARMLSQDTFQVVFDTGSSDLWVPSSKCPSSNIA 71

DEGAL611g00150 ------------------------------------------------------------ 27

DEGAL611g00070 CAKRNRYDAEKSSTYVANGREFNVTYGSGAVRGFLSMDTLSVSGIRVMNQTFAEATWE-S 105

DEGAL611g00060 CAKRNRYDAEKSSTYVANGREFNITYGSGAVRGFLSMDTLSVNGLRVTNQTFAEATWE-S 101

DEGAL611g00090 C-------------------------------GFLSMDTLSVSGLRVMNQTFAEATYE-S 70

DEGAL611g00050 CAQHNRYDAGKSSTYVANGRYFLITYGSGEVEGFLSMDTLSVSGLRVMNQTFAEATSE-S 167

DEGAL611g00020 CLLHNKYHSDKSSTYVKNGTEFKIQYGSGAVSGVLSADTVDLNGMRVTNQTFAEIMRESG 175

DEGAL611g00100 CRTHNKYDAEKSSTHVANGTKFQCIYPLGSISGELSTDTVRVGGLTVTKQTFAEITEE-P 173

DEGAL611g00080 CRTHNKYDAEKSSTHVANGTKFQCIYPLGSISGELSTDTVRVGGLTVTKQTFAEITEE-P 118

HE565350.1 CATHSKYNAEKSSTYVANGTKFAIQYGSGSVSGVLSTDTVSVSGITVTKQTFGEITEESG 102

DEGAL611g00110 CATHSKYNAEKSSTYVANGTKFTIQYGSGSVSGVLSTDTVSVSGITVTKQTFGEITKESG 131

DEGAL611g00150 ----SKYNAEKSSTYVANGTKFAIQYGSGSVSGVLSTDTVSVSGITVTKQTFGEITEESG 83

* ** **: :.*: * :***.* *

DEGAL611g00070 EHFSAKPIDGILGMSYPDLATSGALPVFDQMMAQKVIDKAVFSFYLTRDPKHLPGSELVL 165

DEGAL611g00060 EGFSTDPIDGILGMGYPQLARSRALPVFDQMMAQKVIDKAVFSFYLTRDPKHLTGSELVL 161

DEGAL611g00090 DDFNAEPIDGILGMGYPELATPGALPVFDQMMAQKVIDKAVFSFYLTRDPEHLPGSELVL 130

DEGAL611g00050 KDFSANPIDGILGMGYPELAESRALPVFDQMMAQKVIDKAVFSFYLTRDPKHLPGSELVL 227

DEGAL611g00020 LGFIAGKFDGILGMGYPTIARG-GLPVFDQMVAQNVIDQAVFTFFLTRDPNHPTGSELVL 234

DEGAL611g00100 DAFAYGKYDGILGMGFPEISIS-GLPVFDQMVEQKVVEKAMFSFFITRDQQHPTGSELVL 232

DEGAL611g00080 DAFAYGKYDGILGMGFPEISIS-GLPVFDQMVEQKVVEKAMFSFFITRDQQHPTGSKLVL 177

HE565350.1 DSFIYGKYDGILGMGYPEIASS-GLPVFDQMVKQKVVEKAIFSFFLTRDPQHPIGSELVL 161

DEGAL611g00110 DSFIYGKFDGILGMGYPEIASS-GLPVFDQMVKQKVVEKAIFSFFLTRDPEHPIGSQLVL 190

DEGAL611g00150 DSFIYGKYDGILGMGYPEIASS-GLPVFDQMVKQKVVEKAIFSFFLTRDPQHPIGSELVL 142

* ******.:* :: .*******: *:*:::*:*:*::*** :* **:***

DEGAL611g00070 GGVDRNHYKGKITYVPVTKKKWWEIKMDKLTVDGQTKLHLCKGGCRAIVDSGTSFIVGPV 225

DEGAL611g00060 GGVDRKHYKGKITYVPVTTKQWWEVKMDKLTVDGQTKLHLCKGGCRAIVDSGTSFIVGPF 221

DEGAL611g00090 GGVDRNHYKGKITYVPVTQKKWWEIKMDKLTVDGQTKLHLCKGGCRAIVDSGTSFIVGPV 190

DEGAL611g00050 GGVDRNHYKGKITYVPVTKKKYWEIKMDKLTVDGQTKLHLCKGGCRAIVDSGTSFIVGPV 287

DEGAL611g00020 GGIDPKHHKGEITYTPVTRKGYWQFGVDKIAVSGHSD-ELCKGGCQAIADTGTSLIAGPT 293

DEGAL611g00100 GGIDPNHYKGEITYAPLNVEGYWRFRVDKMTLGGKTA-PVCQNGCHGLVNTGM------- 284

DEGAL611g00080 GGIDPNHYKGEITYAPLNVEGYWRFRVDKMTLGGKTA-PVCQNGCHGLVNTGM------- 229

HE565350.1 GGIDPKHYKGDITYAPLTRESYWQFRVDKVTLNGKAA-PVCQKGCEGIADTGTSLFVGPT 220

DEGAL611g00110 GGIDTKHYKGDITYAPLTREAYWQFRVDTVTLNGKTA-PVCQKGCEGIADTGTSLFVGPT 249

DEGAL611g00150 GGIDPKHYKGDITYAPLTRESYWQFRVDKVTLNGKAA-PVCQKGCEGIADTGTSLFVGPT 201

**:* :*:**.***.*:. : :*.. :*.:::.*:: :*: **..:.::*

DEGAL611g00070 EEAMLLIKTLGGKS-EMGAFTVDCDRASSLPNVEFTIAGKTFKLTPSDYILR-------- 276

DEGAL611g00060 KEAMLLIKTLGGKP-ENDMFTVDCDRASSLPNVEFTIAGKTFELTSSDYILRWSLTGRTA 280

DEGAL611g00090 EEAKLLIKTLGGKR-ENDTYTVDCDRASSLPNVEFTIAGKTFELTSSDYILRWNLTGHTA 249

DEGAL611g00050 EEAKRLIKTLGGKR-ENDTYTVDCDRASSLPNVEFTIAGKTFELTSSDYILRWNLTGHTA 346

DEGAL611g00020 KEVTKLNELIGAAPFIGGEYIVNCKNLPNMPNIEFTISNRTFVLTPDEYILKMSQGSMPV 353

DEGAL611g00100 -YVAALAAQLGAQESASGQYVVDCEKAGSLPNIELAIAGRLFEFTHAEYIVRLNQSSETR 343

DEGAL611g00080 -YVAALAAQLGAQESASGQYVVDCEKAGSLPNIELAIAGRLFEFTHAEYIVRLNQSSETR 288

HE565350.1 ADVAALASQLDAQETAPGLYLVDCEKAGDLPNIEFTIAGRPFELTPLDYVVRLKQSGQTF 280

DEGAL611g00110 ADVAALASQLDAQETAPGLYLVDCEKAGDLPNIEFTIAGRPFELTPLDYVVRIKQSGQTF 309

DEGAL611g00150 ADVAALASQLDAQETAPGLYLVDCEKAGDLPNID----------------------GQTF 239

. * :.. . : *:*.. .:**::

DEGAL611g00070 ----------SYPPQWILGDVMMGKYFTVFDRENDRVGFAEAA*----- 309

DEGAL611g00060 CVLGFLPD-GTNPPLWILGDVMMGKYFTVFDRENDRVGFAEAA*----- 322

DEGAL611g00090 CVLGFAPF-ESYPPLWILGDVMMGKYFTVFDRENDRVVGVVGDSYGVS* 296

DEGAL611g00050 CVLGFAPD-DT--TQWILGDVMMGKYFTVFDRENDRVGFAEAA*----- 386

DEGAL611g00020 CLSGFIGLDVPRDPVWILGDVFIGRYFTVFDRQNDQVGFADAA*----- 396

DEGAL611g00100 CLLAFRGVDIPNFPIWSLGDIFIAKYFTVFDRENHRIGFADAA*----- 386

DEGAL611g00080 CLLAFRGVDIPNFPIWSLGDIFIAKYFTVFDRENHRIGFADAA*----- 331

HE565350.1 CVLAFQGMDIPDDPIWILGDIFIGKYFTVFDRENNRVGFADAA*----- 323

DEGAL611g00110 CVLAFQGMDNPDDPIWILGDIFIGKYFTVFDRENNRVGFADAV*----- 352

DEGAL611g00150 CVLAFQGMDIPDDPIWILGDIFIGKYFTVFDRENNRVGFADAV*----- 282

* ***:::.:*******:*.:: . .

# Percent Identity Matrix - created by Clustal2.1 PROTIEN

#

#

1: DEGAL611g00070 100.00 89.51 87.59 86.64 57.65 53.85 51.06 60.20 56.80 56.07

2: DEGAL611g00060 89.51 100.00 85.22 86.25 59.06 53.85 51.19 59.49 56.27 55.95

3: DEGAL611g00090 87.59 85.22 100.00 91.00 53.98 53.02 50.00 57.50 53.93 53.12

4: DEGAL611g00050 86.64 86.25 91.00 100.00 50.66 51.34 50.77 59.05 54.71 54.51

5: DEGAL611g00020 57.65 59.06 53.98 50.66 100.00 51.96 51.66 61.92 58.97 58.72

6: DEGAL611g00100 53.85 53.85 53.02 51.34 51.96 100.00 99.09 74.84 71.72 74.36

7: DEGAL611g00080 51.06 51.19 50.00 50.77 51.66 99.09 100.00 74.58 72.39 74.39

8: HE565350.1 60.20 59.49 57.50 59.05 61.92 74.84 74.58 100.00 91.33 97.69

9: DEGAL611g00110 56.80 56.27 53.93 54.71 58.97 71.72 72.39 91.33 100.00 95.04

10: DEGAL611g00150 56.07 55.95 53.12 54.51 58.72 74.36 74.39 97.69 95.04 100.00
